# Fis Connects Two Sensory Pathways, Quorum Sensing and Surface Sensing, to Control Motility in *Vibrio parahaemolyticus*

**DOI:** 10.3389/fmicb.2021.669447

**Published:** 2021-10-25

**Authors:** Jessica G. Tague, Abish Regmi, Gwendolyn J. Gregory, E. Fidelma Boyd

**Affiliations:** Department of Biological Sciences, University of Delaware, Newark, DE, United States

**Keywords:** Fis, quorum sensing, motility, metabolism, swarming

## Abstract

Factor for inversion stimulation (Fis) is a global regulator that is highly expressed during exponential phase growth and undetectable in stationary phase growth. Quorum sensing (QS) is a global regulatory mechanism that controls gene expression in response to changes in cell density and growth phase. In *Vibrio parahaemolyticus*, a marine species and a significant human pathogen, the QS regulatory sRNAs, Qrr1 to Qrr5, are expressed during exponential growth and negatively regulate the high cell density QS master regulator OpaR. OpaR is a positive regulator of capsule polysaccharide (CPS) formation, which is required for biofilm formation, and is a repressor of lateral flagella required for swarming motility. In *V. parahaemolyticus*, we show that Fis is a positive regulator of the *qrr* sRNAs expression. In an in-frame *fis* deletion mutant, *qrr* expression was repressed and *opaR* expression was induced. The Δ*fis* mutant produced CPS and biofilm, but swarming motility was abolished. Also, the *fis* deletion mutant was more sensitive to polymyxin B. Swarming motility requires expression of both the surface sensing *scrABC* operon and lateral flagella *laf* operon. Our data showed that in the Δ*fis* mutant both *laf* and *scrABC* genes were repressed. Fis controlled swarming motility indirectly through the QS pathway and directly through the surface sensing pathway. To determine the effects of Fis on cellular metabolism, we performed *in vitro* growth competition assays, and found that Δ*fis* was outcompeted by wild type in minimal media supplemented with intestinal mucus as a sole nutrient source. The data showed that Fis positively modulated mucus components L-arabinose, D-gluconate and N-acetyl-D-glucosamine catabolism gene expression. In an *in vivo* colonization competition assay, Δ*fis* was outcompeted by wild type, indicating Fis is required for fitness. Overall, these data demonstrate a global regulatory role for Fis in *V. parahaemolyticus* that includes QS, motility, and metabolism.

## Introduction

The factor for inversion stimulation (Fis) is a nucleoid associated protein (NAP) that has two major functions in bacteria, chromosome organization and gene regulation (Azam et al., 1999; Ishihama, 2010). Fis, along with other NAPs, is an important regulator of ribosome, tRNA and rRNA expression (Bokal et al., 1997; Auner et al., 2003; Schneider et al., 2003; Dennis et al., 2004). As a transcriptional regulator, it can act as both an activator and repressor of a large number of genes (Keane and Dorman, 2003; Kelly et al., 2004; Browning et al., 2005; Grainger et al., 2006). As an activator, Fis can directly bind to RNA polymerase to affect transcription, or indirectly control transcription *via* DNA supercoiling at promoters (Bokal et al., 1997; McLeod et al., 2002; Auner et al., 2003; Cróinín et al., 2006). Fis controls DNA topology by regulating DNA gyrases (*gyrA* and *gyrB*) and DNA topoisomerase I (*topA*), required for DNA negative supercoiling in *Escherichia coli* and *Salmonella enterica* (Schneider et al., 1999; Keane and Dorman, 2003; Weinstein-Fischer and Altuvia, 2007). In enteric species, Fis was shown to be a global regulator that responded to growth phases and abiotic stresses (González-Gil et al., 1996; Goldberg et al., 2001; Kelly et al., 2004; Browning et al., 2005; Bradley et al., 2007; Lautier and Nasser, 2007; Steen et al., 2010; Zhang et al., 2012; Wang et al., 2013; Lv et al., 2018). In *E. coli*, Fis is one of the most abundant proteins, highly expressed in early exponential phase cells and absent in stationary phase cells, under aerobic growth conditions (Osuna et al., 1995; Mallik et al., 2004). Fis binds to specific sites, however with limited sequence conservation (Finkel and Johnson, 1992). Fis is known as a nucleoid structuring protein that causes DNA bending, allowing for changes in gene expression (Finkel and Johnson, 1992; Bétermier et al., 1994; Skoko et al., 2006). Genome wide studies have shown over a 1,000 binding peaks for Fis in *E. coli* controlling a fifth of chromosomal genes (Grainger et al., 2006; Cho et al., 2008; Kahramanoglou et al., 2011). It has also been shown that many regulatory regions contain multiple and sometimes overlapping Fis binding sites (BS; Hengen et al., 2003; Shao et al., 2008). Genetic, biochemical and structural analysis in *E. coli* of Fis high affinity binding sites has demonstrated the presence of a 15-bp core sequence flanked by G/C (position −7) and C/G (position +7) base-pairs with a central A/T rich region (position 0) (Cho et al., 2008; Shao et al., 2008; Kahramanoglou et al., 2011; Hancock et al., 2016).

In *E. coli* and *S. enterica*, studies have shown that Fis can control virulence, motility, and metabolism (González-Gil et al., 1996; Kelly et al., 2004; Grainger et al., 2006; Bradley et al., 2007). These studies identified 100s of genes whose expression *in vivo* is either enhanced or repressed by Fis. In *S. enterica*, the polar flagellum genes, and genes within several pathogenicity islands, were differentially expressed between a Δ*fis* mutant and wild type (Osuna et al., 1995; Cróinín et al., 2006; Wang et al., 2013). In the plant pathogen, *Dickeya zeae*, a Δ*fis* mutant strain showed a total of 490 genes significantly regulated by Fis (Ouafa et al., 2012; Prigent-Combaret et al., 2012; Lv et al., 2018). In *Vibrio cholerae*, it has been shown that Fis modulates expression of the quorum sensing non-coding regulatory sRNAs (Qrr), *qrr1* to *qrr4* (Lenz and Bassler, 2007). Quorum sensing (QS) is a term used to describe bacterial communication mediate by chemical signals that allows bacteria to control global gene expression in response to cell density changes (Nealson et al., 1970; Fuqua et al., 1994; Gray et al., 1994; Bassler et al., 1997; Bassler, 1999; Miller and Bassler, 2001; Swift et al., 2001; Miller et al., 2002). In *V. cholerae*, it was proposed that Fis acts along with the QS response regulator LuxO, a sigma factor-54 activator, to transcribe the four *qrr1* to *qrr4* sRNAs. The Qrr sRNAs repressed the master QS high cell density (HCD) regulator HapR and activated the QS low cell density (LCD) regulator AphA. In a Δ*fis* mutant in this species, the *qrr* sRNAs were repressed and *hapR* was expressed at wild type levels (Lenz and Bassler, 2007). This is the only other study to examine the role of Fis among *Vibrio* species.

*Vibrio parahaemolyticus* is a marine halophile and the leading cause of bacterial seafood-borne gastroenteritis worldwide with increased incidences of infection due to climate change (Nair et al., 2007; Su and Liu, 2007; Froelich and Noble, 2016; Froelich and Daines, 2020). A *V. parahaemolyticus* infection causes inflammatory diarrhea and its main virulence factors are two type 3 secretions systems and their effector proteins (Makino et al., 2003; O’Boyle and Boyd, 2014; Kodama et al., 2015; De Souza Santos and Orth, 2019; Miller et al., 2019). Unlike *V. cholerae* that only produces a single polar flagellum, *V. parahaemolyticus* produces both a polar flagellum and lateral flagella expressed from the Flh (Fli) and Laf loci, respectively (Belas et al., 1986; McCarter et al., 1988; Stewart and McCarter, 2003). The polar flagellum, required for swimming motility, is produced in cells grown in liquid media and is under the control of sigma factor RpoN (σ54) and its activator FlaK, and sigma factor FliAP (σ28). The lateral flagella, required for swarming motility on solid surfaces, are under the control of RpoN and its activator LafK, and a second σ28 factor FliAL (McCarter and Wright, 1993; Stewart and McCarter, 2003; Jaques and McCarter, 2006; Gode-Potratz et al., 2011; Kernell Burke et al., 2015). Disruption of *rpoN* abolishes all motility, whereas deletion of either of the two σ28 sigma factors, FliAP (FliA) or FliAL, abolishes swimming and swarming, respectively (Stewart and McCarter, 2003; Whitaker et al., 2014). Overall, control of motility in the dual flagellar system of *V. parahaemolyticus* differs significantly from monoflagellar systems of enteric species (Klose and Mekalanos, 1998; McCarter, 1999; Prouty et al., 2001). Previously, it was demonstrated that bacterial motility and metabolism require a functional QS pathway in *V. parahaemolyticus* (Kalburge et al., 2017). In this species, it was shown that deletion of the QS response regulator *luxO* resulted in repression of the five *qrr* genes and constitutive expression of *opaR* (the *hapR* homolog). The Δ*luxO* mutant had reduced swimming motility, but swarming motility was abolished, while a Δ*opaR* mutant was hyper-motile and swarming proficient (Kalburge et al., 2017). The QS regulator OpaR is a direct repressor of the *laf* operon, required for lateral flagellum synthesis and swarming motility (Güvener and McCarter, 2003; Jaques and McCarter, 2006; Gode-Potratz and McCarter, 2011; Kernell Burke et al., 2015). OpaR is an activator of the *cps* operon, required for capsular polysaccharide (CPS) formation, an important component of biofilm (McCarter, 1998; Boles and McCarter, 2002; Kim and McCarter, 2007; Kalburge et al., 2017). Additionally, studies have shown that the *V. parahaemolyticus* surface sensing operon *scrABC* activates swarming motility and represses CPS formation by reducing the intracellular levels of c-di-GMP (Boles and McCarter, 2002; Ferreira et al., 2008; Trimble and McCarter, 2011). A deletion of the *scrABC* operon induces high c-di-GMP levels that repress the *laf* operon and induce *cps* gene expression (Boles and McCarter, 2002; Ferreira et al., 2008; Trimble and McCarter, 2011). The surface colonization regulatory (Scr) program in *V. parahaemolyticus* contains a 100 genes, 70 genes of which are involved in swarming motility and 30 genes involved in biofilm formation that are controlled by intracellular c-di-GMP levels (Boles and McCarter, 2002; Kim and McCarter, 2007; Gode-Potratz and McCarter, 2011; Ferreira et al., 2012). In addition, QS can also modulate c-di-GMP levels to control swarming behavior (Gode-Potratz et al., 2011).

Here, we characterized the role of Fis in *V. parahaemolyticus*, a marine halophile and gastrointestinal pathogen. This work shows that Fis connects the QS and surface sensing signaling pathways in this species to control swarming motility. We determined the expression pattern of *fis* across the growth curve and constructed an in-frame Δ*fis* deletion mutant to examine its role in *V. parahaemolyticus* physiology. For example, the *fis* mutant produced more capsule and was more sensitive to the antimicrobial peptide polymyxin B. We demonstrated a role of Fis in the QS pathway, specifically its modulation of the five non-coding regulatory sRNAs, *qrr1* to *qrr5*, as well as the QS HCD master regulator, OpaR. The effects of a *fis* deletion on swimming and swarming motility were determined and an essential role for Fis in swarming motility was uncovered. DNA binding assays and green fluorescent protein (GFP) reporter assays demonstrated Fis regulation of the *qrr* sRNA genes, the *laf* lateral flagellum operon and the surface sensing *scrABC* operon. We performed *in vitro* growth competition assays and an *in vivo* colonization assay between the Δ*fis* mutant and a *lacZ* knock-in WT strain, WBWlacZ, to demonstrate a fitness effect when *fis* is deleted. Further, our data show that Fis positively modulates the expression of L-arabinose, D-gluconate and N-acetyl-D-glucosamine (NAG) metabolism genes. This study demonstrates that Fis integrates the QS and surface sensing pathways to control swarming motility and is important for control of metabolism and *in vivo* fitness.

## Materials and Methods

### Bacterial Strains, Media, and Culture Conditions

All strains and plasmids used in this study are listed in Supplementary Table S1. A streptomycin-resistant clinical isolate *V. parahaemolyticus* RIMD2210633 was used in this study. Unless stated otherwise, all *V. parahaemolyticus* strains were grown in lysogeny broth (LB) medium (Fischer Scientific, Pittsburgh, PA) containing 3% NaCl (LBS) at 37°C with aeration or M9 minimal media (Sigma Aldrich, St. Louis, MO) supplemented with 3% NaCl (M9S). Antibiotics were added to growth media at the following concentrations: ampicillin (Amp), 100μg/ml, streptomycin (Sm), 200μg/ml, tetracycline (Tet), 1μg/ml, and chloramphenicol (Cm), 12.5μg/ml when required.

### Construction of Δ*fis* Mutant in *V. parahaemolyticus* RIMD2210633

Splicing by overlap extension (SOE) PCR and an allelic exchange method (Ho et al., 1989) were used to construct an in-frame, non-polar deletion mutant of *fis* (VP2885) in *V. parahaemolyticus* RIMD2210633. Briefly, primers were designed using *V. parahaemolyticus* RIMD2210633 genomic DNA as a template. All primers used in this study are listed in Supplementary Table S2. SOE PCR was conducted to obtain an 18bp-truncated version of VP2885 (297-bp). The Δ*fis* PCR fragments were cloned into the suicide vector pDS132 (Philippe et al., 2004) and named pDSΔ*fis*. pDSΔ*fis* was then transformed into *E. coli* strain β2155 λ*pir* (Dehio and Meyer, 1997), and conjugated into *V. parahaemolyticus* RIMD2210633. Conjugation was conducted by cross streaking both strains onto LB plates containing 0.3mm diaminopimelic acid. The colonies were verified for single crossover *via* PCR. The colonies that had undergone a single crossover were grown overnight in LBS with no antibiotic added and plated onto LBS containing 10% sucrose to select for double crossover deletion mutants. The gene deletion was confirmed by PCR and sequencing.

### Phenotype Assays

To observe CPS, heart Infusion media containing 1.5% agar, 2.5mm CaCl_2_, and 0.25% Congo red dye was used and plates were incubated at 30°C, as previously described (Güvener and McCarter, 2003). Biofilm assays were conducted using crystal violet staining. Cultures were grown overnight in LBS and then used to inoculate (1:40 dilution) a 96 well plate, grown static at 37°C. After 24h, the wells are washed with PBS, stained with crystal violet for 30min and then accessed for biofilm formation. The biofilms are then dissolved in DMSO and OD_595_ was measured. Swimming assays were conducted in LB 2% NaCl with 0.6% agar and swarming assays were conducted in heart infusion (HI) media with 2% NaCl and 1.5% agar (Whitaker et al., 2014). To study swimming behavior, a single colony of the bacterium was stabbed into the center of the plate, and plates were incubated at 37°C for 24h. For the swarming assay, plates were spot inoculated on the surface of the media and grown at 30°C for 48h (Whitaker et al., 2014). For polymyxin B sensitivity assays, overnight bacterial cultures were diluted (1:50) into LBS and grown for 2h and then spun down and suspended in 5ml LBS. Polymyxin B sulphate (Sigma-Aldrich) was added to the cultures (final concentration of 40μg/ml) and incubated at 37°C for 1h. For the zero minute time point, an aliquot was obtained before adding the polymyxin B, and then aliquots were taken at 30min and 60min time points. The aliquots were serially diluted and plated to determine the colony forming units (CFUs) at each specific time point. Percent survival was calculated by dividing the CFUs at 30min and 60min with that of 0min and multiplied by 100. For disk assay, similar growth condition as above were used, with 2h growth cultures spread plated onto LBS plates. Polymyxin B disks (100μg of polymyxin in each disk) were placed on each plate and incubated for 24h at 37°C before the zone of inhibitions were measured. Three technical replicates and two biological replicates were performed for each strain.

### Bioinformatics Analysis to Identify Putative Fis Binding Sites

The regulatory region of each gene cluster of interest from *V. parahaemolyticus* RIMD210633 was obtained using NCBI nucleotide database. Virtual footprint was used to identify putative Fis binding sites using the *E. coli* Fis consensus binding sequence (Münch et al., 2003). The 229-bp, 416-bp, 371-bp, 385-bp, 153-bp, 385-bp, and 545bp DNA regions upstream of *flhA* (VP2235-VP2231), *lafB* (VPA1550-VPA1557), *araB* (VPA1674), *nagB* (VPA0038), *gntK* (VP0063), and *scrABC* (VPA1513) respectively, were used as inputs for Fis binding. The regulatory regions of *qrr1* (193-bp), *qrr2* (338-bp), *qrr3* (162-bp), *qrr4* (287-bp), and *qrr5* (177-bp) were also used as inputs. Default settings were used to obtain putative Fis binding sites. A 130-bp sequence of VPA1424 regulatory region was used as a negative control that contains no Fis binding sites. A 229-bp sequence of *gyrA* regulatory region was used as a positive control for Fis binding, which was previously shown to contain Fis binding sites and to be directly regulated by Fis (Schneider et al., 1999; Keane and Dorman, 2003).

### Fis Protein Purification

Fis was purified using a method previously described with modifications as necessary (Carpenter et al., 2015; Kalburge et al., 2017). Briefly, Fis was cloned into the pMAL-c5x expression vector in which a 6X His-tag maltose binding protein (MBP) was fused to *fis* separated by a tobacco etch virus (TEV) protease cleavage site (Liu et al., 2013). Primer pair FisFWDpMAL and FisREVpMAL (Supplementary Table S2) and *V. parahaemolyticus* RIMD2210633 genomic DNA were used to amplify *fis* (VP2885). The *fis* PCR product along with purified pMAL-c5x, were digested with NcoI and BamHI, ligated with T4 ligase, and transformed into DH5α. The vector pMAL-c5x*fis* was purified, sequenced, and then transformed into *E. coli* BL21 (DE3). A 10ml portion of *E. coli* BL21 pMAL-c5x*fis* overnight cultures were used to inoculate 1L of fresh LB supplemented with 100μg/ml ampicillin and 0.2% glucose and grown at 37°C until the OD reached 0.4, at which point, the culture was induced by adding 0.5mm IPTG. The cells were grown overnight at 18°C. Cells were pelleted at 2,800×*g* and resuspended in 15ml of column buffer (50mm sodium phosphate, 200mm NaCl, pH 7.5) supplemented with 0.5mm benzamidine, and 1mm phenylmethylsulphonyl fluoride. Bacterial cells were lysed using a microfluidizer, spun down at 25,000×*g* for 60min, and the supernatant was collected. The supernatant was passed through a 20ml amylose resin (New England BioLabs) and washed with 10 column volumes (CVs) of column buffer. Fis fused with 6X His-MBP was then eluted with three CVs of column buffer supplemented with 20mm maltose. Using 6X His-TEV protease (1:10, TEV:protein in 50mm sodium phosphate, 200mm NaCl, 10mm imidazole, 5mm BME, pH 7.5) the fused protein was cleaved at the TEV cleavage site. The cleaved protein was adjusted to 20mm imidazole and run through an immobilized metal affinity chromatography column using HisPur Ni-NTA resin to remove the cleaved 6X His-MBP and the 6X His-TEV protease. Mass spectrometry was performed to confirm Fis protein molecular weight and SDS-PAGE was conducted to determine its purity.

### Electrophoretic Mobility Shift Assays

The regulatory regions of genes of interest were used as probes in electrophoretic mobility shift assays (EMSA). The regulatory regions of all five *qrr* sRNAs were analyzed for binding of Fis. A 193-bp fragment of P*qrr1*, a 338-bp fragment of P*qrr2*, a 162-bp fragment of P*qrr3*, a 287-bp fragment of P*qrr4*, and a 177-bp fragment of P*qrr5* regulatory regions were used as probes. A 130-bp probe of VPA1424 regulatory region was used as a negative control and a 229-bp probe of P*gyrA* regulatory region was used as a positive control. Analysis of Fis binding to flagellum gene clusters included a 161-bp probe of P*flhA* (VP2235-VP2231) and a 244-bp probe of P*lafB* (VPA1550-VPA1557). The 545-bp regulatory region of *scrABC* (VPA1513-VPA1515) operon was divided into three probes, probe 1147-bp, probe 2136-bp, and probe 3139-bp. For analysis of metabolism, 138-bp and 152-bp probes of P*araB* (VPA1674, L-ribulokinase, L-arabinose catabolism), 154-bp and 120-bp probes of P*nagB* (VPA0038, glucosamine-6-phosphate isomerase, D-glucosamine catabolism), and a138-bp probe of P*gntK* (VP0063, gluconokinase, D-gluconate catabolism) were used in EMSAs. The EMSA probes were PCR amplified using Phusion Hifidelity Polymerase in 50μl reaction mixture using respective primers sets listed in Supplementary Table S2 and *V. parahaemolyticus* RIMD2210633 genomic DNA as template. Various molar ratios of purified Fis were incubated with 30ng of target DNA in binding buffer (10mm Tris, 150mm KCL, 0.1mm dithiothreitol, 0.1mm EDTA, 5% PEG, pH 7.4) for 20min at room temperature. A native acrylamide 6% gel was prepared and pre-run for 2h (200V at 4°C) with 1x Tris-acetate-EDTA (TAE) buffer, and then 10μl of the target DNA-protein mixture was loaded into consecutive lanes. The gel was run at 200V for 2h in 1X TAE buffer at 4°C, which was then stained in an ethidium bromide bath (0.5μg/ml) for 20min and imaged. To examine the specificity of Fis binding sites, first, we used an approach using SOE PCR to create mutations in a Fis BS within the P*nagB* probe 2. Using this approach, we mutated 5 -sites which resulted in less Fis binding, but did not abolish binding since, in this region, there were multiple potential Fis BS. Therefore, for the other regions of interest we used a different approach, designing shorter DNA probes for each regulatory region that contained a single Fis BS. A second mutated probe was also designed to contain mutations at key nucleotide positions (Supplementary Table S3). EMSA were performed on wild type and mutated probes using the same ratios and run on the same gel.

### Transcription Reporter Assays

Green fluorescent protein reporter assays were conducted in *V. parahaemolyticus* RIMD2210633 and Δ*fis* strains. Reporter plasmids were constructed with the regulatory regions of motility genes, *flhA* and *lafB*, and metabolic genes, *araB*, *nagB* and *gntK*, upstream of a promoterless *gfp* gene, as previously described (Gregory et al., 2019). Briefly, primers were designed to amplify the regulatory region upstream of each gene or gene cluster with primer pairs listed in Supplementary Table S2. Each amplified regulatory region was then ligated with the promoterless parent vector pRU1064 (Karunakaran et al., 2005), which had been linearized prior with SpeI, using NEBuilder High Fidelity (HiFi) DNA Assembly Master Mix (New England Biolabs, Ipswich, MA) *via* Gibson Assembly Protocol (Gibson, 2011). Overlapping regions for Gibson Assembly are indicated in lower case letters in the primer sequence in Supplementary Table S2. Reporter plasmid P*_flhA_-gfp* encompasses 269-bp of the regulatory region upstream of *flhA*. Reporter plasmid P*_lafB_-gfp* encompasses 456-bp of the regulatory region upstream of *lafB*. Reporter plasmid P*_araB_-gfp* encompasses 411-bp of the regulatory region upstream of *araB*. Reporter plasmid P*_nagB_-gfp* encompasses 434-bp of the regulatory region upstream of *nagB*. Reporter plasmid P*_gntK_-gfp* encompasses 193-bp of the regulatory region upstream of *gntK*. Reporter plasmid P*_scrABC_-gfp* encompasses 545-bp of the regulatory region upstream of the *scrABC* operon. Additionally, the regulatory region of *qrr1* to *qrr5* were amplified and cloned into the pRU1064 reporter plasmid. The plasmids were transformed into *E. coli* Dh5α, purified and sequenced. Plasmids were then conjugated into wild type and the Δ*fis* mutant for further analysis.

Strains were grown overnight with aeration at 37°C in LBS with Tet (1μg/ml). Cells were then pelleted, washed two times with 1X PBS, and diluted 1:100 in LBS. Strains containing the P*qrr* and P*opaR* reporters were grown to LCD (0.4–0.45 OD), washed two times with 1X PBS, and resuspended to a final OD of 1.0 before measuring relative fluorescence. The metabolism gene reporters were grown for 20h with antibiotic selection. P*_araB_-gfp* was grown in LBS supplemented with 10mm D-arabinose, P*_nagB_-gfp* was grown in LBS supplemented with 10mm D-glucosamine, P*_gntK_-gfp* was grown in LBS supplemented with 10mm D-gluconate. Cells were pelleted and resuspended in 1X PBS. The pRUP*lafB* reporter assay was performed using cells grown on heart infusion (HI) plates for 16h. Colonies were scraped from the plate and resuspended in 1xPBS to a final OD_595_ 0.5. GFP fluorescence was measured with excitation at 385 and emission at 509nm in black, clear-bottom 96-well plates on a Spark microplate reader with Magellan software (Tecan Systems Inc.). Specific fluorescence was calculated for each sample by normalizing relative fluorescence to OD_595_. At least two biological replicates, in triplicate, were performed for each assay. Statistics were calculated using an unpaired Student’s *t*-test.

### *In vitro* Growth Competition Assays

*In vitro* growth competition assays were performed by diluting an inoculum 1:50 into LBS broth, and separately in M9S supplemented with mouse intestinal mucus or 10mm of individual carbon sources, D-glucose, L-arabinose, L-ribose, D-gluconate, D-glucosamine, and NAG. A β-galactosidase knock in *V. parahaemolyticus* RIMD2210633 strain, WBWlacZ, which was previously shown to grow similarly to wild type *in vitro* and *in vivo*, was used for all the competition assays (Whitaker et al., 2012, 2014). The culture was incubated at 37°C for 24h, serially diluted and plated on LBS plus streptomycin and 5-bromo-4-chloro-3-indolyl-B-D-galactoside (X-gal). The competitive index (CI) was determined using the following equation: CI=ratio out (Δ*fis*/WBWlacZ)/ratio in (Δ*fis*/WBWlacZ). A CI of <1 indicates WBWlacZ outcompetes the Δ*fis* mutant, a CI of >1 indicates that the Δ*fis* mutant outcompetes WBWlacZ. The ratio of Δ*fis* to WBWlacZ in the inoculum mixture is termed as “Ratio in” and the ratio of Δ*fis* to WBWlacZ colonies recovered from the mouse intestine is referred as “Ratio out.”

### *In vivo* Colonization Competition Assays

All mice experiments were approved by the University of Delaware Institutional Animal Care and Use Committee (Whitaker et al., 2012, 2014). Inoculum for competition assays was prepared using overnight cultures of WBWlacZ and Δ*fis* diluted into fresh LBS media and grown for 4h. Exponential phase cultures were then pelleted by centrifugation at 4,000×*g*, washed and resuspended in PBS. One ml of WBWlacZ and one ml of Δ*fis* were prepared, corresponding to 1×10^10^CFU of each strain, based on the previously determined OD and CFU ratio. A 500μl aliquot of Δ*fis* was combined with 500μl of the WBWlacZ, yielding a total bacterial concentration of 1×10^10^CFU/ml. The inoculum was serially diluted and plated on LBS agar plate supplemented with 200μg/ml streptomycin and 8μg/ml of X-gal to determine the exact ratio of the inoculum. Male C57BL/6 mice aged 6 to 10weeks were housed under specific-pathogen-free conditions in standard cages in groups (5 per group) and provided standard mouse feed and water *ad libitum*. Pretreatment of mice with streptomycin was performed as previously described (Whitaker et al., 2012, 2014; Haines-Menges et al., 2014; Kalburge et al., 2017). Mice were inoculated with 100μl of the bacterial suspension and 24h post-infection, mice were sacrificed, and the entire gastrointestinal tract was harvested. Samples were placed in 8ml of sterile 1x PBS, mechanically homogenized and serially diluted in 1xPBS. Diluted samples were plated for CFUs on LBS, supplemented with streptomycin and X-gal for a blue (WBWlacZ) versus white (Δ*fis*) screen of colonies after incubation at 37°C overnight. The competitive index (CI) was determined as described above.

## Results

### *fis* Expression Is Controlled in a Growth Dependent Manner

In *V. parahaemolyticus* RIMD2210633, locus tag VP2885 is annotated as a Fis protein homolog, a 98 amino acid protein that shows 100% protein identity to Fis from *V. cholerae* and 82% amino acid identity to Fis from *E. coli*. Fis is an abundant protein in *E. coli*, highly expressed in exponential phase cells and undetected in stationary phase cells grown under aerobic conditions (Azam et al., 1999; Mallik et al., 2004). In *V. parahaemolyticus* RIMD2210633, we determined the expression pattern of *fis* across the growth curve, *via* RNA isolated from wild type cells grown in LBS at 37°C aerobically at various optical densities (ODs). Using quantitative real time PCR analysis, *fis* showed highest expression levels in exponential cells at Ods 0.15, 0.25, and 0.5 and then rapidly declined at Ods 0.8 and 1.0, as cells entered stationary phase (Supplementary Figure S1). These data show that *fis* in *V. parahaemolyticus* has a similar expression pattern to *fis* in *E. coli* and also what has been demonstrated in *V. cholerae* (Azam et al., 1999; Mallik et al., 2004; Lenz and Bassler, 2007).

### Fis Positively Regulates the QS Non-coding *qrr* sRNAs

**Figure 1:**
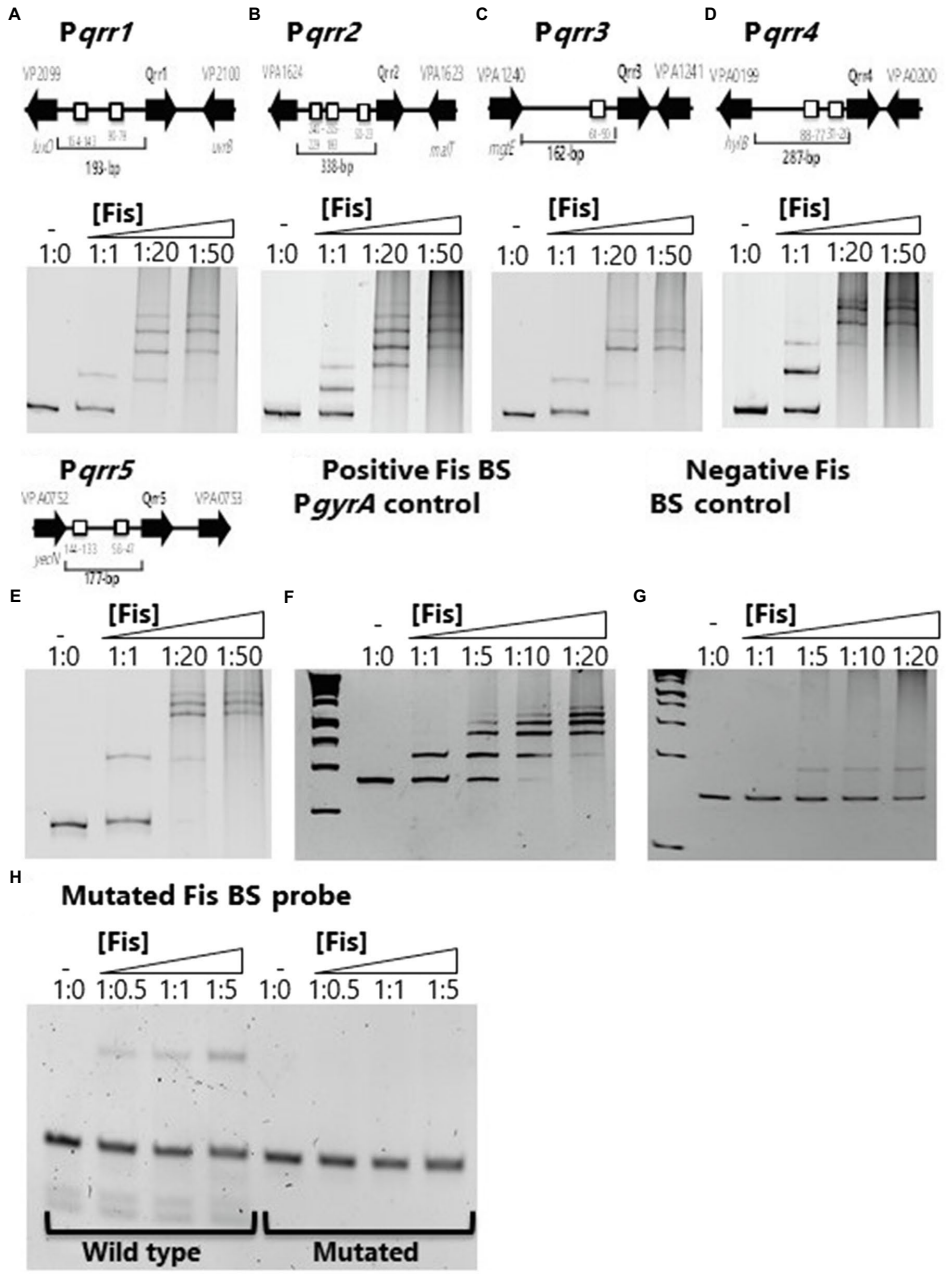
Factor for inversion stimulation binds to the regulatory regions of *V. parahaemolyticus* regulatory sRNAs **(A)** *qrr1*, **(B)** *qrr2*, **(C)** *qrr3*, **(D)** *qrr4*, and **(E)** *qrr5*. A putative Fis binding site (BS) is shown as a box in the regulatory region of *qrr1* to *qrr5*. Electrophoretic mobility shift assays (EMSA) using purified Fis and the regulatory regions of *qrr1* to *qrr5*. DNA:protein ratios are as follows: 1:0, 1:1, 1:20, 1:50. **(F)** Positive Fis BS control in *gyrA* regulatory region showing complete shift at all concentrations. **(G)** Fis non-binding negative control using DNA with no putative Fis BS showing weak non-specific binding and no shift at lowest concentration. **(H)** *qrr3* probe containing a single Fis BS (wild type) and a mutated BS demonstrating specificity of Fis binding.

One other study has examined the role of Fis in *Vibrio* and this work showed that in *V. cholerae* Fis was a positive regulator of the QS non-coding regulatory sRNA genes *qrr1* to *qrr4* (Lenz and Bassler, 2007). The *qrr1* to *qrr4* genes in *V. parahaemolyticus* were homologous to those present in *V. cholerae* and showed identical genome locations in both species for each *qrr* gene. To determine the role of Fis in the regulation of *qrr1* to *qrr5* in *V. parahaemolyticus*, we identified multiple putative Fis binding sites within the regulatory regions of all five sRNAs (Figure 1). To demonstrate Fis binding, we purified the Fis protein and constructed DNA probes of the regulatory region of each *qrr* sRNA and performed EMSA with increasing concentrations of purified Fis. Fis binding was shown in a concentration dependent manner in all five P*qrr-*Fis EMSAs that resulted in a complete shift of the probe (Figures 1A–E). Smearing in the wells at higher Fis concentrations was noted but was not present in a protein only control lane. Slight smearing in the lane at the highest ratios of DNA:protein, is potentially due to multimerization of Fis protein that can occur, but we cannot rule out minute DNA contamination. The regulatory region of *gyrA* was used as a Fis binding positive control and showed specific concentration dependent binding with a complete shift in the probe in all five *qrr* genes (Figure 1F). A DNA fragment without a putative Fis binding site was used as a non-binding control and showed non-specific, weak binding, and no complete shift of the probe at any concentration (Figure 1G). To examine the specificity of binding further, we aligned the putative Fis binding sites in the regulatory regions of the *qrr* genes, most of which had multiple and sometimes overlapping binding sites. However, we identified a single Fis binding motif in the coding region of *qrr3* and mutated this conserved sites to examine Fis binding. In these EMSAs, no banding shift was observed in the mutated probe (Figure 1H).

**Figure 2:**
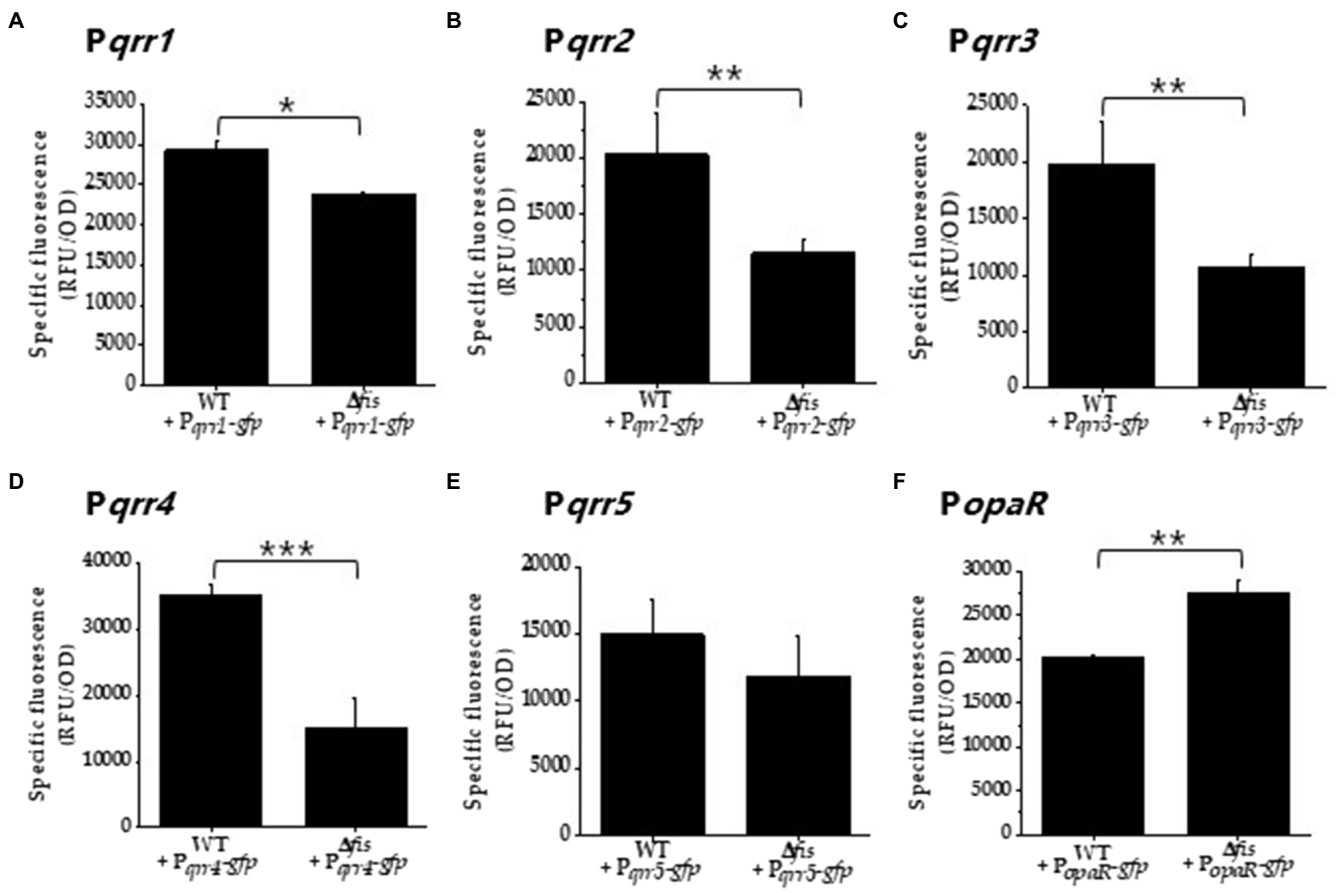
Fis is a positive regulator of the *qrr* genes. **(A)** P*qrr1*, **(B)** P*qrr2*, **(C)** P*qrr3*, **(D)** P*qrr4*, **(E)** P*qrr5*, and **(F)** P*opaR* green fluorescent protein (GFP) transcriptional reporter assays in wild type and the Δ*fis* mutant in cultures grown to OD 0.4–0.45 and measured for specific fluorescence (RFU/OD). Means and standard deviations of three biological replicates are plotted. Statistics calculated using a Student’s *t*-test (^*^p<0.05, ^**^p<0.01, ^***^p<0.001).

To further examine the role of Fis in *V. parahaemolyticus qrr* gene expression, an in-frame deletion of *fis* was constructed by deleting 279-bp of VP2885. We examined growth of the Δ*fis* mutant in LBS broth and found that it grew identical to wild type *V. parahaemolyticus* (Supplementary Figure S2A). However, on LBS agar plates, the Δ*fis* mutant formed a small colony morphology compared to wild type. This phenotype was rescued by complementation with a functional copy of *fis* under the control of an IPTG inducible promoter, restoring wild type colony morphology (Supplementary Figures S2B,C). Next, we performed transcriptional GFP reporter assays using the regulatory region of each *qrr* to determine whether deletion of *fis* affects expression. Cells were grown to 0.4–0.45 OD and GFP levels measured using relative fluorescence normalized to OD (specific fluorescence). In Δ*fis*, the overall expression of P*_qrr2_*-*gfp* to P*_qrr4_*-*gfp* was significantly downregulated compared to wild type (*p*<0.01 and 0.001, respectively), indicating that Fis is a positive regulator of *qrr2*, *qrr3*, and *qrr4* in *V. parahaemolyticus* (Figures 2B–D). We observed a reduction in P*_qrr1_*-*gfp* and P*_qrr5_*-*gfp* expression in the Δ*fis* mutant relative to wild type, but this reduction was less significant, which may not be physiologically relevant (Figures 2A,E). Next, we examined whether *opaR* expression was changed in the Δ*fis* mutant using GFP reporter expression assays under the control of the *opaR* regulatory region. In these assays, P*_opaR_*-*gfp* activity was significantly upregulated (*p*<0.01) in the Δ*fis* mutant compared to wild type (Figure 2F). Overall, the data suggest that Fis plays a role in the QS pathway, but is not an essential component, instead it likely modulates expression of the *qrr* sRNAs and *opaR* in early exponential phase cells. The data also suggest that other factors are required to control *qrr* expression. For example, studies have shown that, AphA, LuxO, OpaR, and LuxT also significantly control *qrr* expression in *Vibrio* species (Rutherford et al., 2011; Zhang et al., 2012; Kalburge et al., 2017; Eickhoff et al., 2021; Simpson et al., 2021).

### Fis Controls Multiple Phenotypes in *V. parahaemolyticus*

**Figure 3:**
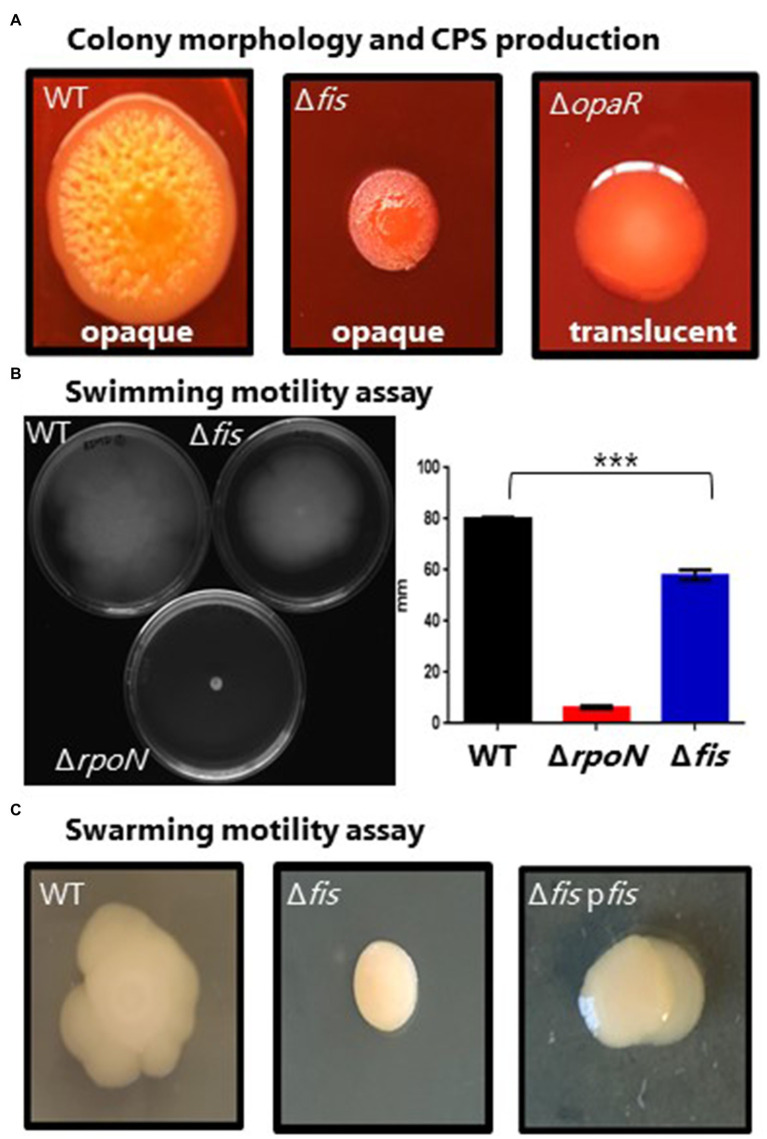
Phenotypic analysis of the Δ*fis* mutant. **(A)** Capsule polysaccharide (CPS) production in wild type (WT), Δ*fis* and Δ*opaR* mutants was observed after incubation for 48h at 30°C. Images are an example of three biological replicates performed in triplicate. **(B)** Swimming motility assays and quantification. Two biological replicates were measured, performed in triplicate. Statistics calculated using a Student’s *t*-test (^***^p<0.001). **(C)** Swarming motility assay of *V. parahaemolyticus* wild type and the Δ*fis* mutant and Δ*fis* complementation strain. Complementation assays were grown in the presence of Cm and IPTG. All images are examples from three biological replicates.

OpaR, the QS master regulator, is a positive regulator of CPS required for biofilm formation. We examined the effects of a *fis* deletion on this phenotype given the changes in expression of *opaR* in the Δ*fis* mutant. CPS production in *V. parahaemolyticus* manifests as an opaque rough wrinkly colony morphology also known as rugose morphology that is also an indirect measure of c-di-GMP levels that oppositely control CPS and swarming motility (McCarter, 1998; Boles and McCarter, 2002; Trimble and McCarter, 2011; Ferreira et al., 2012; Jones and Wozniak, 2017). In the wild-type strain on Congo red plates, cells formed large opaque wrinkly raised colonies, whereas a Δ*opaR* mutant formed a large smooth colony morphology, indicating CPS is lacking (Figure 3A). The Δ*fis* mutant produced a much smaller highly wrinkly colony morphology (Figure 3A). This colony morphology is indicative of overproduction of CPS in bacteria (Jones and Wozniak, 2017).

**Figure 4:**
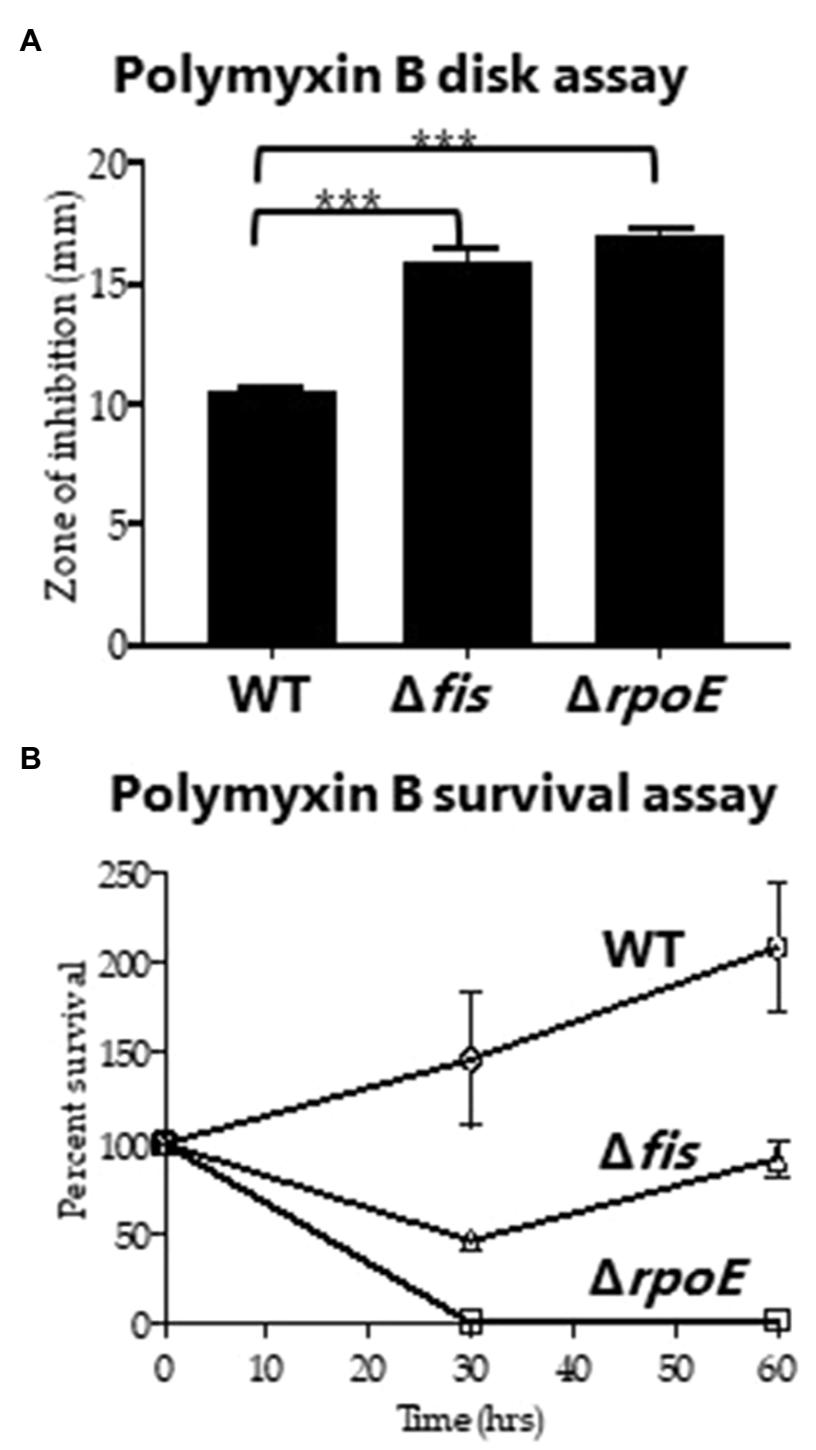
Polymyxin B sensitivity assay. **(A)** Disk containing 100μg of total polymyxin B was used to identify the zone of inhibition for WT, Δ*fis* and Δ*rpoE* and was quantified by measuring the diameter of the zone of inhibition. **(B)** Survival assays were conducted using WT, Δ*fis*, and Δ*rpoE* after treating the bacterial cultures with polymyxin B (final concentration of 40μg/ml) and calculating percent survival at 30min and 60min. The disk assay was performed in duplicate using two biological replicates. The survival assay was performed in triplicate using two biological replicates. Unpaired *t*-test was conducted to determine the value of *p*. ^***^p<0.001.

To investigate whether Fis has any role in antimicrobial peptide resistance, the Δ*fis* strain was examined for polymyxin B sensitivity using disk diffusion and survival assays. Previous work in *Vibrio* has shown that changes in the bacterial cell wall can result in increased sensitivity to polymyxin B (Haines-Menges et al., 2014; Lubin et al., 2015; McDonald et al., 2018). We used Δ*rpoE* as a control, which lacks the sigma factor RpoE that is required for the cell envelope stress response and was previously shown to be polymyxin B sensitive (Haines-Menges et al., 2014). For the disk diffusion assay, the Δ*fis* and Δ*rpoE* mutants had significantly larger zones of inhibition compared to wild type indicating these mutants are more sensitive to polymyxin B (Figure 4A). To verify this further, we conducted survival assay using 200μg of total polymyxin B in 5ml of LBS media. CFUs were counted at 0, 30 and 60min post exposure. Significantly lower CFUs for the Δ*fis* were recovered at 30 and 60min compared to wild type (Figure 4B). The data demonstrated that Fis is required for polymyxin B resistance in *V. parahaemolyticus*, although the mechanism remains unknown.

Next, we examined whether deletion of *fis* affected motility in *V. parahaemolyticus*, a species that produces both polar and lateral flagella, and where OpaR is a negative regulator of the *laf* operon (Jaques and McCarter, 2006). Swimming assays demonstrated that the Δ*fis* mutant had a defect in motility compared to wild type with reduced spreading across the agar plate (Figure 3B). An Δ*rpoN* mutant was also examined as a negative control, and this mutant, as expected, showed no motility (Figure 3B). In swarming assays, the wild-type strain produced a typical swarming cauliflower colony morphology, whereas in the Δ*fis* deletion mutant, swarming motility was abolished suggesting lateral flagella are absent (Figure 3C). Complementation of the Δ*fis* mutant with a functional copy of the *fis* gene under the control of an IPTG-inducible promoter rescued swarming and swimming motility (Supplementary Figure S3).

### Fis Is Necessary for Motility in *V. parahaemolyticus*

**Figure 5:**
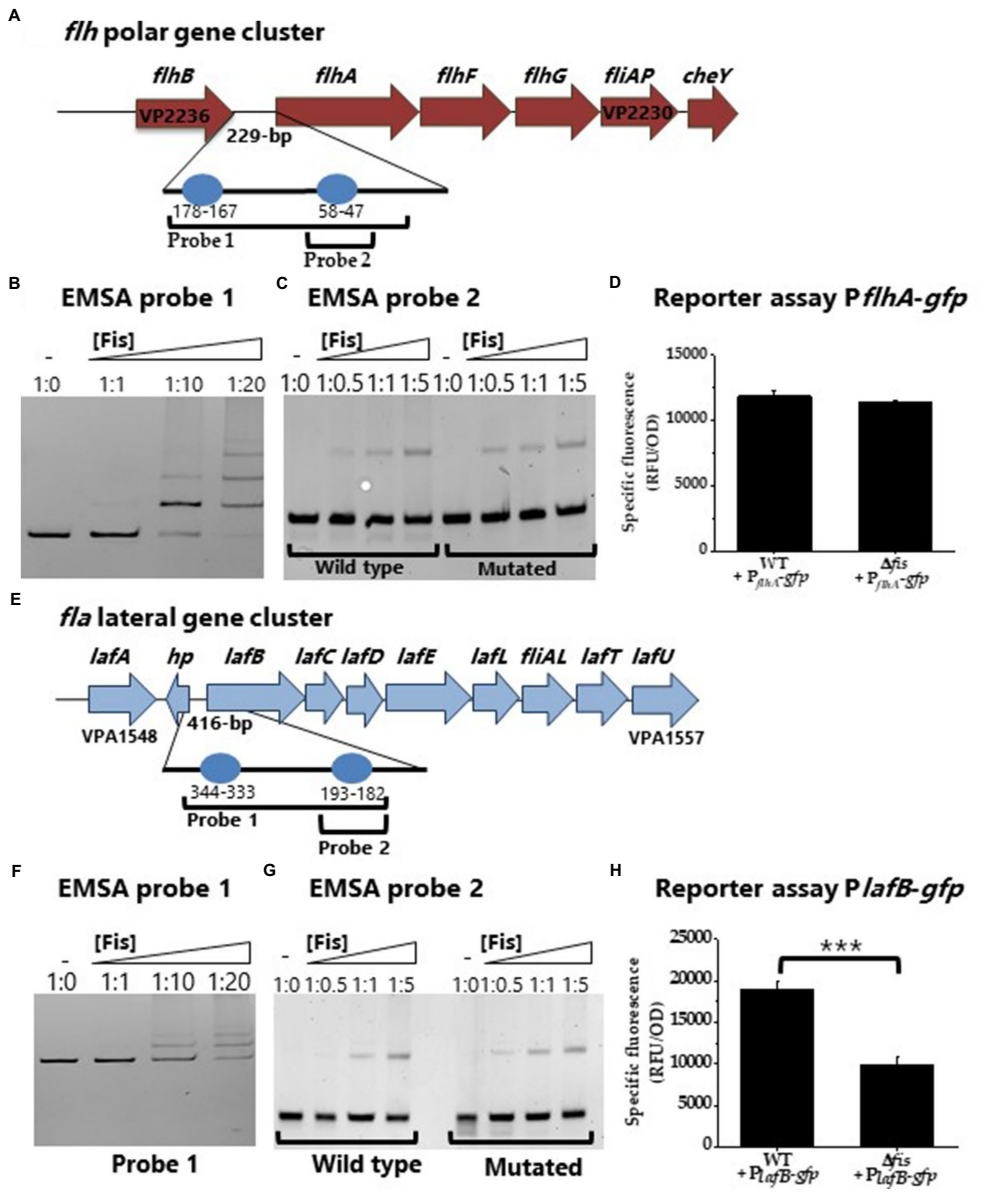
Regulation of lateral flagella biosynthesis by Fis. **(A)** Regulatory region of polar flagellum *flh* genes with putative Fis binding sites (BS) depicted as blue circles. **(B)** EMSA of Fis bound to P*flhA* in a concentration dependent manner. DNA: protein ratios are as follows: 10, 1:1, 1:10, 1:20. **(C)** Probe 2 containing a single Fis BS (WT) was mutated. DNA: protein ratios are as follows: 0, 1:0.5, 1:1, 1:5. **(D)** Transcriptional GFP reporter assay of P*_flhA_*-*gfp* in the Δ*fis* mutant relative to wild type. **(E)** Regulatory region of lateral flagellum *laf* genes with putative Fis binding sites. **(F)** EMSA of Fis bound to P*laf* DNA probe. **(G)** Probe 2 containing a single Fis BS and a mutated probe 2 Fis BS. DNA: protein ratios are as follows: 0, 1:0.5, 1:1, 1:5. **(H)** Transcriptional GFP reporter assay of P*_lafB_*-*gfp* between wild type and Δ*fis* (^***^p<0.001).

To further investigate Fis control of swimming and swarming motility, using bioinformatics, we identified multiple putative Fis binding sites within the regulatory regions of both the polar and lateral flagella biosynthesis operons (Figures 5A,E). We performed EMSA analysis using purified Fis and DNA probes of the regulatory regions of the polar flagellum operon (*flh* loci VP2235-PV2231) and the lateral flagellum operon (*laf* loci VPA1550-VPA1557) and demonstrated binding (Figures 5B,F). To determine the specificity of Fis binding further, we created two DNA probes with mutations in a single Fis binding motif and examined binding. In these EMSAs, the wild type sequence showed weak binding for both wild type probes and no change in the mutated probe (Figures 5C,G). These data suggest that Fis binds at these sites in a non-specific manner. To examine Fis regulation of motility further, GFP reporter expression assays were performed. In these assays, the Δ*fis* mutant relative to wild type did not show differential expression of P*_flhA_*-*gfp* (Figure 5D). In contrast, in reporter assays of swarming cells, P*_lafB_*-*gfp* was significantly repressed (*p*<0.001) in the Δ*fis* mutant relative to wild type (Figure 5H). Thus, the data suggest that Fis controls lateral flagella gene expression and is an important positive regulator of swarming motility.

### Fis Is a Positive Regulator of the Surface Sensing Operon *scrABC*

**Figure 6:**
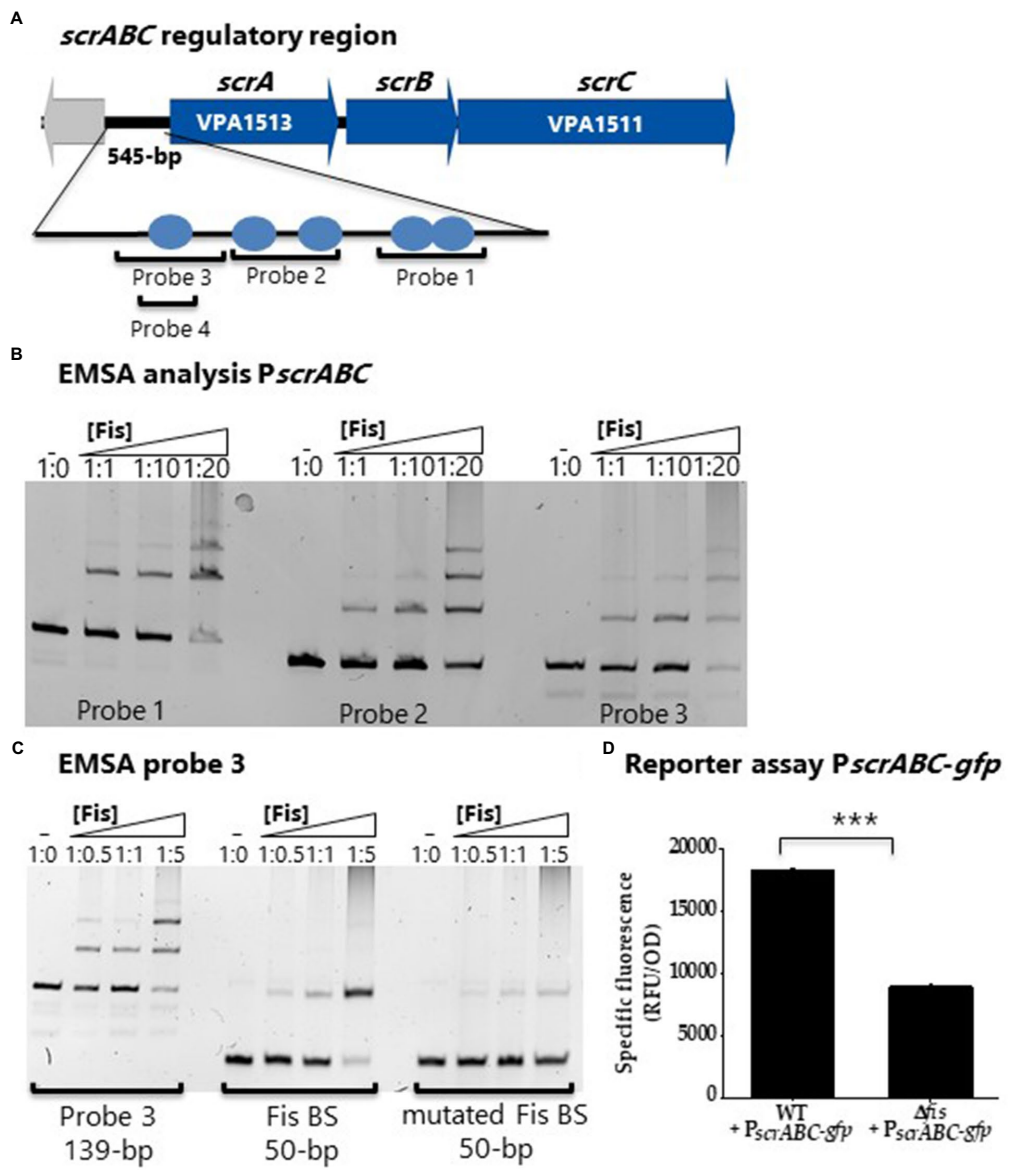
Fis is a positive regulator of the surface sensing operon, *scrABC*. **(A)** Putative Fis binding sites identified in the regulatory region of the *scrABC* surface sensing operon. **(B)** EMSA using purified Fis protein and probes 1, 2 and 3 encompassing the regulatory region of *scrABC*. DNA: protein ratios are as follows: 10, 1:1, 1:10, 1:20, 1:50. **(C)** Probe 3 containing a single Fis BS and a mutated probe 3 Fis BS. DNA: protein ratios are as follows: 0, 1:0.5, 1:1, 1:5. **(D)** GFP reporter assay of P*_scrABC_*-*gfp* in wild type and the Δ*fis* mutant. Specific fluorescence was calculated (RFU/OD) for three biological replicates and plotted as mean and standard deviation. Statistics were calculated using a Student’s *t*-test (^***^p<0.001).

The *scrABC* operon has been shown to positively control swarming motility and negatively control CPS by controlling c-di-GMP levels (Boles and McCarter, 2002; Kim and McCarter, 2007; Gode-Potratz et al., 2011; Ferreira et al., 2012). We reasoned that Fis might also regulate this operon to co-ordinate with the QS pathway in the control of this phenotype. First, using bioinformatics, we identified multiple and sometimes overlapping putative Fis binding sites in the regulatory region of the *scrABC* operon (Figure 6A). We performed EMSAs with three probes that contained Fis BS and demonstrated Fis binding to all three probes in a concentration dependent manner (Figure 6B). To show Fis BS specificity, we created a DNA probe from probe 3 that contains one Fis BS, mutated this site, and performed an EMSA. In this EMSA, Fis still bound to the mutated probe, but at a reduced level (Figure 6C). In GFP reporter assays, P*_scrABC_*-*gfp* activity was significantly downregulated (*p*<0.001) in the Δ*fis* mutant compared to wild type, indicating that Fis is a direct positive regulator of this operon (Figure 6D). Overall, the data suggest that loss of swarming motility is likely due to Fis direct regulation of the *scrABC* operon and the lateral flagella biosynthesis *laf* operon and indirect regulation of *opaR* through modulation of *qrr* expression in *V. parahaemolyticus*.

### Fis Modulates Expression of Carbon Catabolism Genes

**Figure 7:**
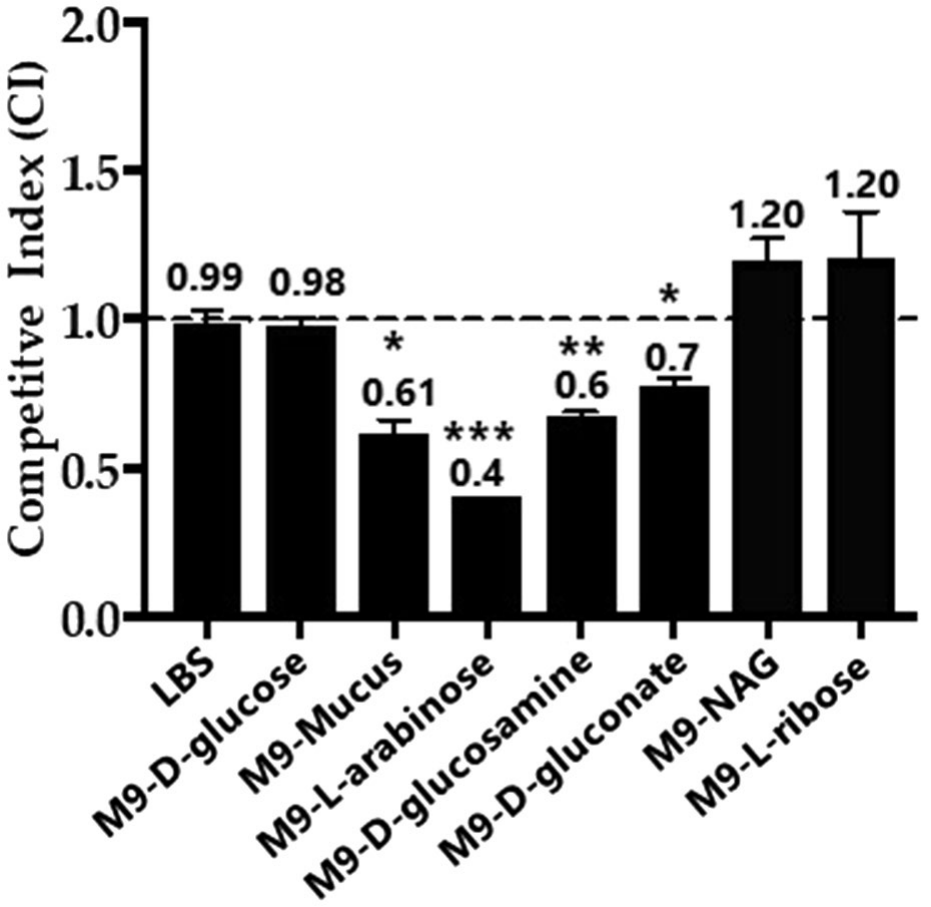
*In vitro* growth competition assay between wild type and the Δ*fis* mutant. WBWlacZ and Δ*fis* were grown in co-culture (1:1 ratio) for 24h in LBS, M9 supplemented with 100μg/ml intestinal mucus, 10mm of D-glucose, L-arabinose, D-glucosamine, D-gluconate, N-acetyl-D-glucosamine (NAG), or D-ribose. WBWlacZ outcompetes Δ*fis*, <1.00, and Δ*fis* outcompetes WBWlacZ when >1.00. The assay was conducted in two biological replicates in triplicates. Error bar indicates SEM. Unpaired Student’s *t*-test was conducted. The significant difference is denoted by asterisks (^*^p<0.05, ^**^p<0.01, ^***^p<0.001).

To determine the effects of Fis on overall metabolism, we conducted growth competition assays between wild type and Δ*fis* in various carbon sources. For the *in vitro* competition assays, we used a β-galactosidase knock-in strain of RIMD2210633, strain WBWlacZ, which was previously demonstrated to behave identically to wild type in *in vitro* and *in vivo* studies (Whitaker et al., 2012, 2014; Haines-Menges et al., 2014; Kalburge et al., 2017). Comparisons of WBWlacZ and the Δ*fis* mutant allows for a blue/white colony screen in the presence of IPTG. First, *in vitro* growth competition assays were performed in LB 3% NaCl (LBS) resulting in a competitive Index (CI) of 0.99 demonstrating under these conditions that neither strain had a competitive advantage. Similarly, *in vitro* competition assays in M9 3%NaCl (M9S) supplemented with glucose showed neither strain had a competitive advantage. However, in M9S supplemented with mouse intestinal mucus or individual mucus components as sole carbon sources, Δ*fis* was significantly outcompeted by WBWlacZ. In mouse intestinal mucus, the Δ*fis* mutant had a CI of 0.61, and in mucus components L-arabinose a CI of 0.4, NAG a CI of 0.68 and D-gluconate a CI of 0.78 (Figure 7).

**Figure 8:**
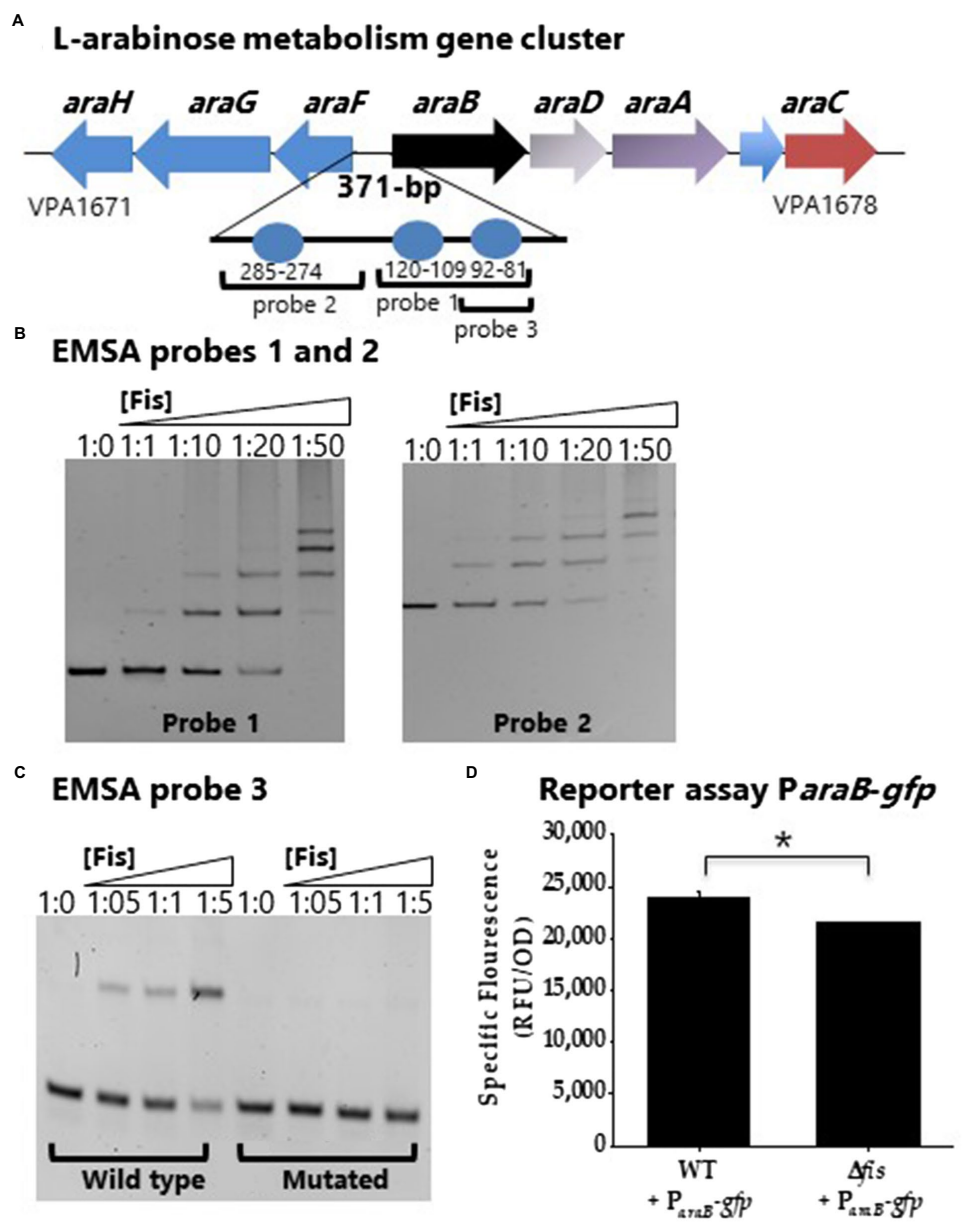
Fis is a positive regulator of the L-arabinose operon *araBCDA*. **(A)** Fis binding sites identified in the regulatory region of the *araBDAC* operon depicted as blue circles. **(B)** P*araB* was divided into two probes, probe 1 and probe 2 for EMSA analysis using purified Fis protein. **(C)** EMSA analysis probe 3 wild type with a single Fis BS and a mutated probe 3. DNA: protein ratios were as follows: 10, 1:1, 1:10, 1:20, 1:50. **(D)** GFP transcriptional reporter assay of the *araBDAC* regulatory region in wild type and the Δ*fis* mutant. ^*^p<0.05.

Putative Fis binding sites were identified in the regulatory region of the *araBDAC* (L-arabinose catabolism) operon (Figure 8A). EMSAs were performed using 138-bp and 152-bp DNA probes containing two and one putative Fis binding sites, respectively (Figure 8A). In these assays, Fis bound to the regulatory region of *araBDAC* and the binding was concentration dependent (Figure 8B). To examine the specificity of Fis binding further, we created probe 3 that contained a single Fis BS, mutated this site, and performed an EMSA. The wild type probe 3 showed Fis binding, whereas the mutated probe showed no binding, demonstrating Fis binding specificity (Figure 8C). In GFP transcriptional reporter assays, P*_araB_*-*gfp* showed lower activity in the Δ*fis* mutant compared to wild type, although the fold change difference is small suggesting it may not be physiologically relevant at this time-point (Figure 8D).

**Figure 9:**
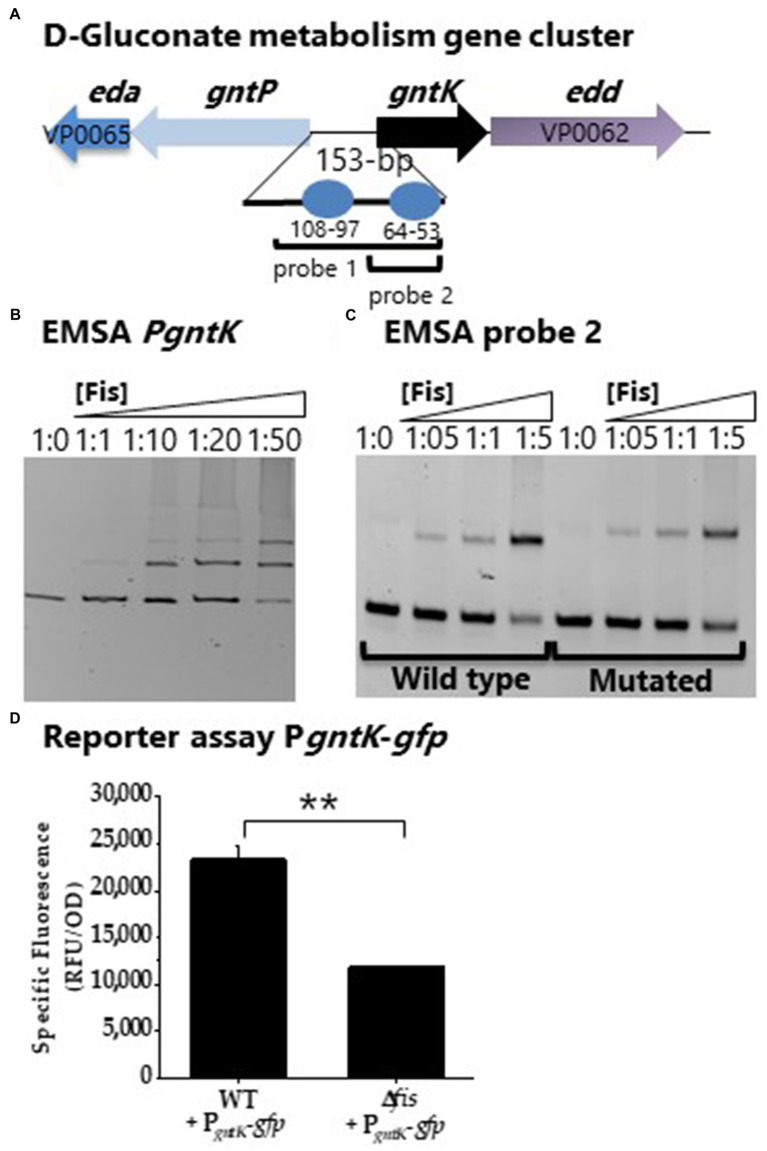
Fis is a positive regulator of *gntK*. **(A)** Putative Fis binding sites in the regulatory region of *gntK*. **(B)** EMSA analysis with purified Fis protein and PgntK probe 1. DNA: protein ratios were as follows: 10, 1:1, 1:10, 1:20, 1:50. **(C)** EMSA analysis of probe 2 containing a single Fis BS and a mutated Fis BS probe 2. **(D)** GFP transcriptional reporter assay of *gntK* regulatory region in wild type and Δ*fis*. ^**^p<0.01.

Factor for inversion stimulation binding sites were also identified in the regulatory region of *gntK* (D-gluconate catabolism; Figure 9A). A DNA probe encompassing P*gntK* showed Fis binding (Figure 9B). A second probe with only a single putative Fis BS was constructed and the putative Fis BS was mutated and EMSAs performed. In this assay a slight reduction in binding is noted, but non-specific binding cannot be ruled out (Figure 9C). To examine Fis control of D-gluconate metabolism further, a GFP reporter assay of P*gntK* was performed. In these assays, P*gntK* showed significantly lower expression levels in the Δ*fis* mutant compared to wild type indicating that Fis is a positive regulator of expression (Figure 9D).

**Figure 10:**
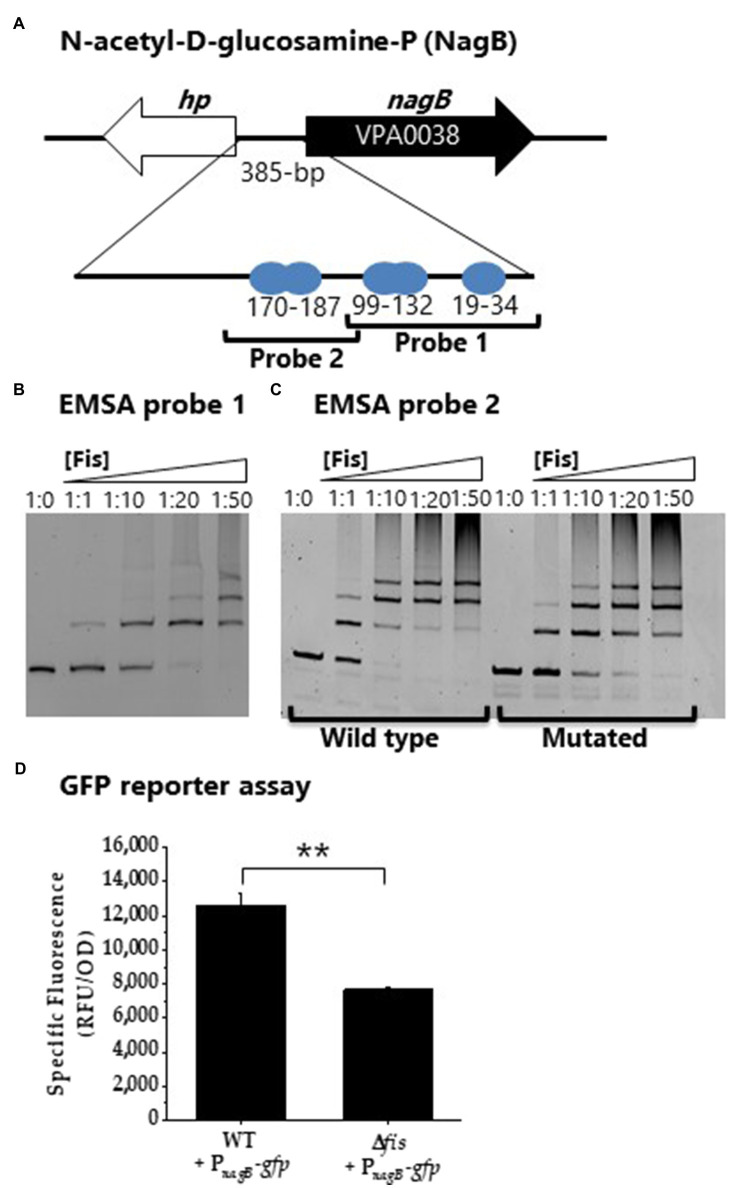
Fis is a positive regulator of NAG gene *nagB*. **(A)** Fis binding sites identified in the regulatory region of *nagB*. **(B)** EMSA analysis of Fis binding to probe 1. **(C)** EMSA analysis of probe 2 and a mutated Fis BS probe 2. DNA: protein molar ratios were as follows: 10, 1:1, 1:10, 1:20, 1:50. **(D)** GFP transcriptional reporter assay with P*_nagB_*-*gfp*. Means and standard deviations of two biological replicates are shown. Statistics calculated using a Student’s *t*-test (^**^p<0.01).

Multiple overlapping Fis binding sites were identified in the regulatory region of *nagB* (NAG catabolism; Figure 10A). EMSA analysis with two probes encompassing the Fis sites showed specific binding in a concentration dependent manner (Figures 10B,C). To examine specificity of binding further we created a probe 2 with mutations in 5 sites of the Fis BS motif. In this analysis, Fis bound to the mutated probe, but the binding was reduced, suggesting Fis is a direct regulator of *nagB* (Figure 10C). In addition, P*nagB* showed significantly lower activity in the GFP reporter assay in the Δ*fis* mutant compared to wild type (Figure 10D). Overall, our results demonstrated that Fis is a positive regulator of catabolism genes, *araB*, *gntK*, and *nagB* in *V. parahaemolyticus*.

**Figure 11:**
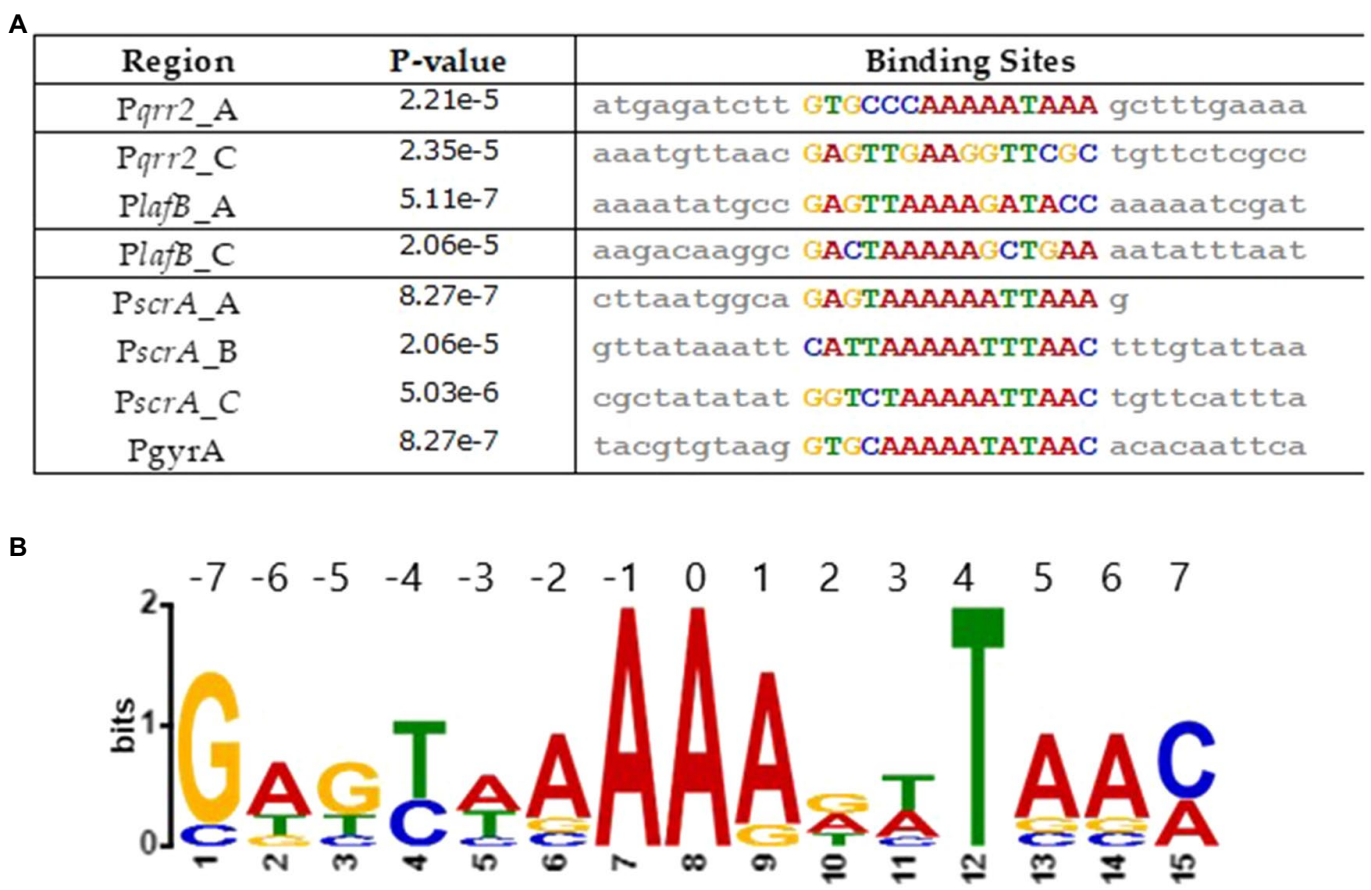
Analysis of Fis consensus sequence. **(A)** A Fis DNA binding motif was created using MEME analysis. Sequences containing a putative Fis binding site were used. **(B)** An alignment of the motifs found in each sequence, along with the corresponding values of *p*.

We determined a Fis consensus sequence from the regulatory regions of genes under Fis control. To accomplish this, we examined a 50-bp region encompassing each putative Fis binding site identified in this study using multiple EM for motif elicitation (MEME) analysis. This analysis identified a 15-bp core motif highly similar to what has been identified in *E. coli* (Figure 11A). This motif is aligned in each sequence and maps to the Fis binding sites identified *via* virtual footprint (Figure 11B). Similar to the sequence motif used by virtual footprint, along with previous reports of Fis consensus sequences, the motif we present has a stretch of T/A bps, characteristic of Fis binding (Cho et al., 2008).

### Fis Is Required for *in vivo* Fitness in *V. parahaemolyticus*

To determine whether Fis contributes to *in vivo* fitness of *V. parahaemolyticus*, *in vivo* colonization competition assays were performed using a streptomycin pretreated adult mouse model of colonization (Whitaker et al., 2012, 2014; Haines-Menges et al., 2014; Yang et al., 2019). The *in vivo* competition assay determined the ability of WBWlacZ and the Δ*fis* mutant to co-colonize the intestinal tract of streptomycin pretreated adult mice. Competition assays were performed in adult C57BL/6 mice pretreated with an orogastric dose of streptomycin (20mg/animal) 24h prior to orogastric co-inoculation with a 1:1 mixture of *V. parahaemolyticus* WBWlacZ and Δ*fis* (*n*=9). In an *in vitro* assay in LBS using the same inoculum, the WBWlacZ vs. Δ*fis* had a CI of 1.17 whereas *in vivo* the WBWlacZ vs. Δ*fis* CI was 0.57 indicating that the mutant was significantly (*p*<0.01) outcompeted by WBWlacZ (Supplementary Figure S4). This indicates that Δ*fis* has decreased fitness *in vivo* compared to the wild type strain.

## Discussion

Nucleoid associated proteins such as Fis, bind and bend DNA to aid in DNA compaction, and are also important global regulators of gene expression. In *V. parahaemolyticus* we show, similar to enteric species, that *fis* is expressed in early to mid-exponential phase cells and declines in late exponential and stationary phase cells. The only other study examining the role of Fis in a *Vibrio* species, showed a direct role of Fis in the QS pathway in *V. cholerae*. They demonstrated Fis activation of *qrr1* to *qrr4* sRNAs and *hapR* (*opaR* homolog) constitutive expression in a *fis* deletion mutant (Lenz and Bassler, 2007). In *V. parahaemolyticus*, Fis bound to all five *qrr* sRNAs regulatory regions in a concentration dependent manner. Our work showed that Fis is a positive regulator of the QS regulatory sRNAs. We show that in the *fis* deletion mutant *qrr1* to *qrr5* were repressed and *opaR* was induce. It is of interest to note that in our study, the expression of each of the *qrr* sRNA differed in the Δ*fis* mutant compared to wild type with significant repression of *qrr2*, *qrr3* and *qrr4*, whereas both *qrr1* and *qrr5* showed reduced expression. The importance of this remains to be determine since we do not know whether in *V. parahaemolyticus* the Qrr sRNAs are redundant as is the case in *V. cholerae* or additive as is the case in *V. harveyi*. Our data suggest that other regulators are also involved in modulating *qrr* expression and that Fis is one of several factors controlling expression. This is not too surprising as studies have shown that, although Fis may bind to a large number of regulatory regions, only a portion of these sites are significantly regulated by Fis (Kahramanoglou et al., 2011; Monteiro et al., 2020). For example, in *E. coli*, ChIP-seq analysis uncovered 1,464 Fis binding sites, but only 462 genes were differential regulated by Fis under the conditions examined (Kahramanoglou et al., 2011). This suggests that Fis has a regulatory role, however other factors are likely involved and that the Fis regulon varies with changes in growth conditions, growth phase, binding affinity, amongst other factors.

**Figure 12:**
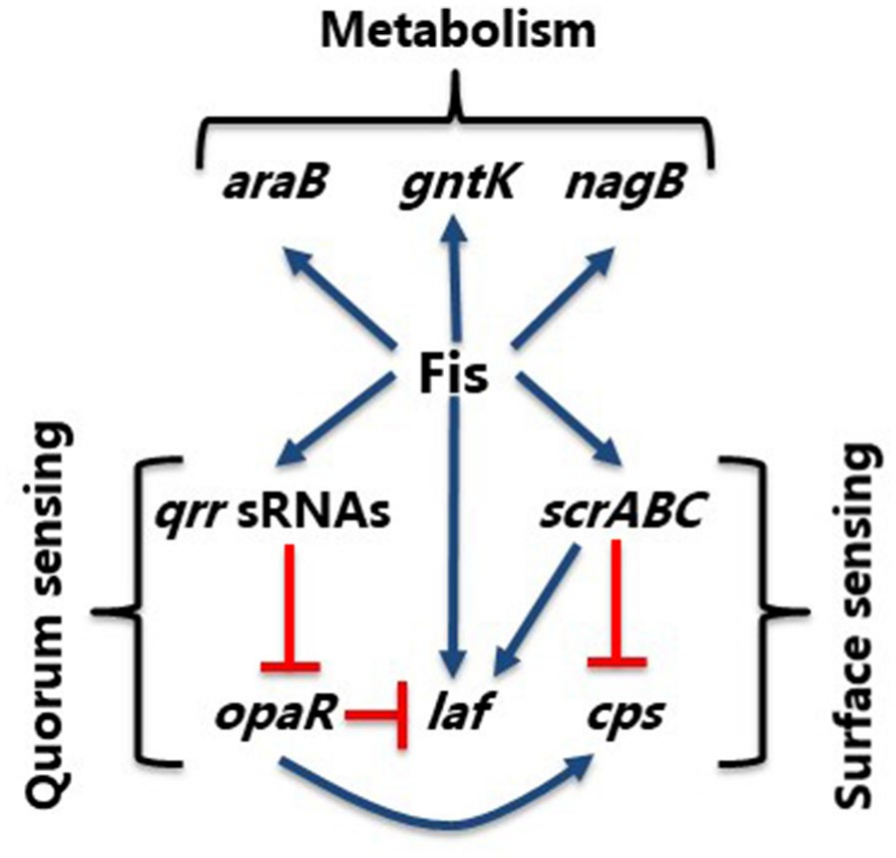
Model of Fis integration of QS and surface sensing pathways. Model shows control of swarming (*laf*) and CPS (*cps*) production in *V. parahaemolyticus*. Blue arrows show positive regulation and orange hammers show negative regulation.

In *V. parahaemolyticus*, we showed that Fis is a positive regulator of swarming motility through modulated expression of the QS regulator, OpaR, and control of the lateral flagellum *laf* operon and the surface sensing *scrABC* operon. The *scrABC* operon in *V. parahaemolyticus* controls the transition between swarming motility and adhesion to a surface by altering gene expression of the *laf* and *cps* operons (Boles and McCarter, 2002; Kim and McCarter, 2007). Together, ScrA, ScrB, and ScrC modulate the level of c-di-GMP in the cell, a secondary messenger that controls numerous downstream processes. More specifically, ScrC contains both GGDEF and EAL enzymatic activity, making it a unique bifunctional enzyme (Kim and McCarter, 2007; Ferreira et al., 2012). ScrA produce the extracellular S-signal, which represses CPS gene expression. In the presence of ScrA interaction with ScrB, ScrC acts as a phosphodiesterase to degrade c-di-GMP. High levels of c-di-GMP promote CPS production, while low levels of c-di-GMP promote swarming motility (Trimble and McCarter, 2011; Ferreira et al., 2012). In the Δ*fis* mutant, we observed repression of both the *laf* and *scrABC* operons in the GFP reporter assays, indicating that Fis is a positive regulator of these operons. We suggest that the Δ*fis* mutant swarming defect is through repression of both the *laf* and *scrABC* operons and through derepression of *opaR*, which is a repressor of swarming motility (Figure 12). As shown in Supplementary Figure S1, *fis* expression is induced at early exponential phase cells and decreases as the cells transition to HCD. We propose that at LCD, Fis activates the *qrr* sRNAs, which repress *opaR*, and activates the surface sensing operon. The *scrABC* operon activates the *laf* operon, stimulating motility, while repressing the *cps* operon. As the cells transition to HCD, *fis* expression decreases while *opaR* expression increases, as it has been shown to be maximally expressed in stationary phase cells. OpaR represses the *laf* operon and activates expression of the *cps* operon, inducing CPS formation. Therefore, Fis integrates both the QS and surface sensing signals by positively regulating *qrr* sRNAs and *scrABC* to induce swarming motility and repress CPS formation until the cells enter HCD (Figure 12).

In *E. coli*, using global approaches, Fis was shown to regulate genes involved in, but not limited to, two-component systems, biofilm formation, and pilus organization. It was determined that Fis regulates genes required for sugar metabolism as well, including NAG as shown in our study (Gawade et al., 2020). In *S. enterica*, Fis was shown to negatively regulate genes contributing to virulence and metabolism in the mammalian gut (Kelly et al., 2004). In this species, it was demonstrated that Fis was a negative regulator of acetate metabolism, biotin synthesis, fatty acids metabolism and propanediol utilization, amongst others. The authors speculated that this could be important for intestinal colonization and/or systemic infection (Kelly et al., 2004). In *V. parahaemolyticus*, the *in vivo* competition assays between the Δ*fis* and WBWLacZ strain showed that the Δ*fis* strain was outcompeted demonstrating that Fis is required for *in vivo* fitness. In addition, *in vitro* growth competition assays in intestinal mucus and mucus components demonstrated that Δ*fis* was again outcompeted by wild type. We speculate that the Δ*fis* mutant is outcompeted by wild type *in vivo* at least in part due to its inability to efficiently utilize nutrient sources. Carbon metabolism was previously implicated as important for colonization of *V. parahaemolyticus* in a streptomycin pretreated adult mouse model (Whitaker et al., 2014; Kalburge et al., 2017). Additionally, we showed that Fis is important for resistance to antimicrobial peptides prevalent in animal intestines. The *fis* deletion mutant was highly sensitive to polymyxin B, which could also account for its reduced fitness *in vivo*. In summary, Fis modulates expression of genes involved in QS, motility, and metabolism in *V. parahaemolyticus* and it will be of interest to determine the different mechanisms used to modulate expression by this NAP.

## Data Availability Statement

The original contributions presented in the study are included in the article/Supplementary Material, further inquiries can be directed to the corresponding author.

## Ethics Statement

The animal study was reviewed and approved by University of Delaware Institutional Animal Care and Use Committee.

## Author Contributions

EB, JT, and AR designed the study and analyzed the results. JT, GG, and AR performed the experiments. All authors contributed to writing and review the manuscript and approved the submitted version.

## Funding

This research was supported in part by a National Science Foundation grant (award IOS-1656688) to EB. JT and GG were funded by University of Delaware graduate fellowship awards.

## Conflict of Interest

The authors declare that the research was conducted in the absence of any commercial or financial relationships that could be construed as a potential conflict of interest.

## Publisher’s Note

All claims expressed in this article are solely those of the authors and do not necessarily represent those of their affiliated organizations, or those of the publisher, the editors and the reviewers. Any product that may be evaluated in this article, or claim that may be made by its manufacturer, is not guaranteed or endorsed by the publisher.

## References

[ref1] Auner H. Buckle M. Deufel A. Kutateladze T. Lazarus L. Mavathur R.. (2003). Mechanism of transcriptional activation by FIS: role of core promoter structure and DNA topology. J. Mol. Biol. 331, 331–344. doi: 10.1016/S0022-2836(03)00727-7, PMID: 12888342

[ref2] Azam T. A. Iwata A. Nishimura A. Ueda S. Ishihama A. (1999). Growth phase-dependent variation in protein composition of the *Escherichia coli* nucleoid. J. Bacteriol. 181, 6361–6370. doi: 10.1128/JB.181.20.6361-6370.1999, PMID: 10515926 PMC103771

[ref3] Bassler B. (1999). How bacteria talk to each other: regulation of gene expression by quorum sensing. Curr. Opin. Microbiol. 2, 582–587. doi: 10.1016/S1369-5274(99)00025-9, PMID: 10607620

[ref4] Bassler B. L. Greenberg E. P. Stevens A. M. (1997). Cross-species induction of luminescence in the quorum-sensing bacterium *Vibrio harveyi*. J. Bacteriol. 179, 4043–4045. doi: 10.1128/jb.179.12.4043-4045.1997, PMID: 9190823 PMC179216

[ref5] Belas R. Simon M. Silverman M. (1986). Regulation of lateral flagella gene transcription in *Vibrio parahaemolyticus*. J. Bacteriol. 167, 210–218. doi: 10.1128/jb.167.1.210-218.1986, PMID: 3013835 PMC212863

[ref6] Bétermier M. Galas D. Chandler M. (1994). Interaction of Fis protein with DNA: bending and specificity of binding. Biochimie 76, 958–967. doi: 10.1016/0300-9084(94)90021-3, PMID: 7748940

[ref7] Bokal A. J. Ross W. Gaal T. Johnson R. C. Gourse R. L. (1997). Molecular anatomy of a transcription activation patch: FIS-RNA polymerase interactions at the *Escherichia coli* rrnB P1 promoter. EMBO J. 16, 154–162. doi: 10.1093/emboj/16.1.154, PMID: 9009276 PMC1169622

[ref8] Boles B. R. McCarter L. L. (2002). *Vibrio parahaemolyticus* scrABC, a novel operon affecting swarming and capsular polysaccharide regulation. J. Bacteriol. 184, 5946–5954. doi: 10.1128/JB.184.21.5946-5954.2002, PMID: 12374828 PMC135390

[ref9] Bradley M. D. Beach M. B. de Koning A. P. Pratt T. S. Osuna R. (2007). Effects of Fis on *Escherichia coli* gene expression during different growth stages. Microbiology 153, 2922–2940. doi: 10.1099/mic.0.2007/008565-0, PMID: 17768236

[ref10] Browning D. F. Grainger D. C. Beatty C. M. Wolfe A. J. Cole J. A. Busby S. J. (2005). Integration of three signals at the *Escherichia coli* nrf promoter: a role for Fis protein in catabolite repression. Mol. Microbiol. 57, 496–510. doi: 10.1111/j.1365-2958.2005.04701.x, PMID: 15978080

[ref11] Carpenter M. Rozovsky S. Boyd E. (2015). Pathogenicity island cross talk mediated by recombination directionality factors facilitates excision from the chromosome. J. Bacteriol. 198, 766–776. doi: 10.1128/JB.00704-15, PMID: 26668266 PMC4810611

[ref12] Cho B. Knight E. Barrett C. Palsson B. (2008). Genome-wide analysis of Fis binding in *Escherichia coli* indicates a causative role for A-/AT-tracts. Genome Res. 18, 900–910. doi: 10.1101/gr.070276.107, PMID: 18340041 PMC2413157

[ref13] Cróinín T. O. Carroll R. K. Kelly A. Dorman C. J. (2006). Roles for DNA supercoiling and the Fis protein in modulating expression of virulence genes during intracellular growth of *Salmonella enterica* serovar Typhimurium. Mol. Microbiol. 62, 869–882. doi: 10.1111/j.1365-2958.2006.05416.x, PMID: 16999831

[ref14] De Souza Santos M. Orth K. (2019). The role of the type III secretion system in the intracellular lifestyle of enteric pathogens. Microbiol. Spectr. 7: doi: 10.1128/microbiolspec.BAI-0008-2019, PMID: 31152523 PMC11026088

[ref15] Dehio C. Meyer M. (1997). Maintenance of broad-host-range incompatibility group P and group Q plasmids and transposition of Tn5 in *Bartonella henselae* following conjugal plasmid transfer from *Escherichia coli*. J. Bacteriol. 179, 538–540. doi: 10.1128/jb.179.2.538-540.1997, PMID: 8990308 PMC178726

[ref16] Dennis P. P. Ehrenberg M. Bremer H. (2004). Control of rRNA synthesis in *Escherichia coli*: a systems biology approach. Microbiol. Mol. Biol. Rev. 68, 639–668. doi: 10.1128/MMBR.68.4.639-668.2004, PMID: 15590778 PMC539008

[ref17] Eickhoff M. Fei C. Huang X. Bassler B. (2021). LuxT controls specific quorum-sensing-regulated behaviors in *Vibrionaceae* spp. via repression of qrr1, encoding a small regulatory RNA. PLoS Genet. 17:e1009336. doi: 10.1371/journal.pgen.1009336, PMID: 33793568 PMC8043402

[ref18] Ferreira R. B. Antunes L. C. Greenberg E. P. McCarter L. L. (2008). *Vibrio parahaemolyticus* ScrC modulates cyclic dimeric GMP regulation of gene expression relevant to growth on surfaces. J. Bacteriol. 190, 851–860. doi: 10.1128/JB.01462-07, PMID: 17993539 PMC2223563

[ref19] Ferreira R. B. Chodur D. M. Antunes L. C. Trimble M. J. McCarter L. L. (2012). Output targets and transcriptional regulation by a cyclic dimeric GMP-responsive circuit in the *Vibrio parahaemolyticus* Scr network. J. Bacteriol. 194, 914–924. doi: 10.1128/JB.05807-11, PMID: 22194449 PMC3294815

[ref20] Finkel S. Johnson R. (1992). The Fis protein: it’s not just for DNA inversion anymore. Mol. Microbiol. 6, 3257–3265. doi: 10.1111/j.1365-2958.1992.tb02193.x, PMID: 1484481

[ref21] Froelich B. Daines D. (2020). In hot water: effects of climate change on vibrio-human interactions. Environ. Microbiol. 22, 4101–4111. doi: 10.1111/1462-2920.14967, PMID: 32114705

[ref22] Froelich B. Noble R. (2016). Vibrio bacteria in raw oysters: managing risks to human health. Philos. Trans. R. Soc. Lond. Ser. B Biol. Sci. 371:20150209. doi: 10.1098/rstb.2015.0209, PMID: 26880841 PMC4760139

[ref23] Fuqua W. C. Winans S. C. Greenberg E. P. (1994). Quorum sensing in bacteria: the LuxR-LuxI family of cell density-responsive transcriptional regulators. J. Bacteriol. 176, 269–275. doi: 10.1128/jb.176.2.269-275.1994, PMID: 8288518 PMC205046

[ref24] Gawade P. Gunjal G. Sharma A. Ghosh P. (2020). Reconstruction of transcriptional regulatory networks of Fis and H-NS in *Escherichia coli* from genome-wide data analysis. Genomics 112, 1264–1272. doi: 10.1016/j.ygeno.2019.07.013, PMID: 31356968

[ref25] Gibson D. G. (2011). Enzymatic assembly of overlapping DNA fragments. Methods Enzymol. 498, 349–361. doi: 10.1016/b978-0-12-385120-8.00015-2, PMID: 21601685 PMC7149801

[ref26] Gode-Potratz C. J. Kustusch R. J. Breheny P. J. Weiss D. S. McCarter L. L. (2011). Surface sensing in *Vibrio parahaemolyticus* triggers a programme of gene expression that promotes colonization and virulence. Mol. Microbiol. 79, 240–263. doi: 10.1111/j.1365-2958.2010.07445.x, PMID: 21166906 PMC3075615

[ref27] Gode-Potratz C. J. McCarter L. L. (2011). Quorum sensing and silencing in *Vibrio parahaemolyticus*. J. Bacteriol. 193, 4224–4237. doi: 10.1128/JB.00432-11, PMID: 21705592 PMC3147687

[ref28] Goldberg M. D. Johnson M. Hinton J. C. Williams P. H. (2001). Role of the nucleoid-associated protein Fis in the regulation of virulence properties of enteropathogenic *Escherichia coli*. Mol. Microbiol. 41, 549–559. doi: 10.1046/j.1365-2958.2001.02526.x, PMID: 11532124

[ref29] González-Gil G. Bringmann P. Kahmann R. (1996). FIS is a regulator of metabolism in *Escherichia coli*. Mol. Microbiol. 22, 21–29. doi: 10.1111/j.1365-2958.1996.tb02652.x, PMID: 8899705

[ref30] Grainger D. C. Hurd D. Goldberg M. D. Busby S. J. (2006). Association of nucleoid proteins with coding and non-coding segments of the *Escherichia coli* genome. Nucleic Acids Res. 34, 4642–4652. doi: 10.1093/nar/gkl542, PMID: 16963779 PMC1636352

[ref31] Gray K. M. Passador L. Iglewski B. H. Greenberg E. P. (1994). Interchangeability and specificity of components from the quorum-sensing regulatory systems of *Vibrio fischeri* and *Pseudomonas aeruginosa*. J. Bacteriol. 176, 3076–3080. doi: 10.1128/jb.176.10.3076-3080.1994, PMID: 8188610 PMC205467

[ref32] Gregory G. J. Morreale D. P. Carpenter M. R. Kalburge S. S. Boyd E. F. (2019). Quorum sensing regulators AphA and OpaR control expression of the operon responsible for biosynthesis of the compatible solute ectoine. Appl. Environ. Microbiol. 85, 1543–1519. doi: 10.1128/aem.01543-19, PMID: 31519665 PMC6821967

[ref33] Güvener Z. McCarter L. (2003). Multiple regulators control capsular polysaccharide production in *Vibrio parahaemolyticus*. J. Bacteriol. 185, 5431–5441. doi: 10.1128/JB.185.18.5431-5441.2003, PMID: 12949095 PMC193756

[ref34] Haines-Menges B. Whitaker W. B. Boyd E. F. (2014). Alternative sigma factor RpoE is important for *Vibrio parahaemolyticus* cell envelope stress response and intestinal colonization. Infect. Immun. 82, 3667–3677. doi: 10.1128/IAI.01854-14, PMID: 24935982 PMC4187813

[ref35] Hancock S. Stella S. Cascio D. Johnson R. (2016). DNA sequence determinants controlling affinity, stability and shape of DNA complexes bound by the nucleoid protein Fis. PLoS One 11:e0150189. doi: 10.1371/journal.pone.0150189, PMID: 26959646 PMC4784862

[ref36] Hengen P. Lyakhov I. Stewart L. Schneider T. (2003). Molecular flip-flops formed by overlapping Fis sites. Nucleic Acids Res. 31, 6663–6673. doi: 10.1093/nar/gkg877, PMID: 14602927 PMC275571

[ref37] Ho S. N. Hunt H. D. Horton R. M. Pullen J. K. Pease L. R. (1989). Site-directed mutagenesis by overlap extension using the polymerase chain reaction. Gene 77, 51–59. doi: 10.1016/0378-1119(89)90358-2, PMID: 2744487

[ref38] Ishihama A. (2010). Prokaryotic genome regulation: multifactor promoters, multitarget regulators and hierarchic networks. FEMS Microbiol. Rev. 34, 628–645. doi: 10.1111/j.1574-6976.2010.00227.x, PMID: 20491932

[ref39] Jaques S. McCarter L. (2006). Three new regulators of swarming in *Vibrio parahaemolyticus*. J. Bacteriol. 188, 2625–2635. doi: 10.1128/JB.188.7.2625-2635.2006, PMID: 16547050 PMC1428401

[ref40] Jones C. Wozniak D. (2017). Congo red stain identifies matrix overproduction and is an indirect measurement for c-di-GMP in many species of bacteria. Methods Mol. Biol. 1657, 147–156. doi: 10.1007/978-1-4939-7240-1_12, PMID: 28889292

[ref41] Kahramanoglou C. Seshasayee A. S. N. Prieto A. I. Ibberson D. Schmidt S. Zimmermann J.. (2011). Direct and indirect effects of H-NS and Fis on global gene expression control in *Escherichia coli*. Nucleic Acids Res. 39, 2073–2091. doi: 10.1093/nar/gkq934, PMID: 21097887 PMC3064808

[ref42] Kalburge S. S. Carpenter M. R. Rozovsky S. Boyd E. F. (2017). Quorum sensing regulators are required for metabolic fitness in *Vibrio parahaemolyticus*. Infect. Immun. 85, 930–916. doi: 10.1128/iai.00930-16, PMID: 28069817 PMC5328491

[ref43] Karunakaran R. Mauchline T. H. Hosie A. H. Poole P. S. (2005). A family of promoter probe vectors incorporating autofluorescent and chromogenic reporter proteins for studying gene expression in Gram-negative bacteria. Microbiology 151, 3249–3256. doi: 10.1099/mic.0.28311-0, PMID: 16207908

[ref44] Keane O. M. Dorman C. J. (2003). The gyr genes of *Salmonella enterica* serovar Typhimurium are repressed by the factor for inversion stimulation, Fis. Mol. Gen. Genomics. 270, 56–65. doi: 10.1007/s00438-003-0896-1, PMID: 12898222

[ref45] Kelly A. Goldberg M. D. Carroll R. K. Danino V. Hinton J. C. D. Dorman C. J. (2004). A global role for Fis in the transcriptional control of metabolism and type III secretion in *Salmonella enterica* serovar Typhimurium. Microbiology 150, 2037–2053. doi: 10.1099/mic.0.27209-0, PMID: 15256548

[ref46] Kernell Burke A. Guthrie L. T. C. Modise T. Cormier G. Jensen R. V. McCarter L. L.. (2015). OpaR controls a network of downstream transcription factors in *Vibrio parahaemolyticus* BB22OP. PLoS One 10:e0121863. doi: 10.1371/journal.pone.0121863, PMID: 25901572 PMC4406679

[ref47] Kim Y. K. McCarter L. L. (2007). ScrG, a GGDEF-EAL protein, participates in regulating swarming and sticking in *Vibrio parahaemolyticus*. J. Bacteriol. 189, 4094–4107. doi: 10.1128/JB.01510-06, PMID: 17400744 PMC1913424

[ref48] Klose K. E. Mekalanos J. J. (1998). Distinct roles of an alternative sigma factor during both free-swimming and colonizing phases of the *Vibrio cholerae* pathogenic cycle. Mol. Microbiol. 28, 501–520. doi: 10.1046/j.1365-2958.1998.00809.x, PMID: 9632254

[ref49] Kodama T. Hiyoshi H. Okada R. Matsuda S. Gotoh K. Iida T. (2015). Regulation of *Vibrio parahaemolyticus* T3SS2 gene expression and function of T3SS2 effectors that modulate actin cytoskeleton. Cell. Microbiol. 17, 183–190. doi: 10.1111/cmi.12408, PMID: 25495647

[ref50] Lautier T. Nasser W. (2007). The DNA nucleoid-associated protein Fis co-ordinates the expression of the main virulence genes in the phytopathogenic bacterium *Erwinia chrysanthemi*. Mol. Microbiol. 66, 1474–1490. doi: 10.1111/j.1365-2958.2007.06012.x, PMID: 18028311

[ref51] Lenz D. H. Bassler B. L. (2007). The small nucleoid protein Fis is involved in *Vibrio cholerae* quorum sensing. Mol. Microbiol. 63, 859–871. doi: 10.1111/j.1365-2958.2006.05545.x, PMID: 17181781

[ref52] Liu J. Li F. Rozovsky S. (2013). The intrinsically disordered membrane protein selenoprotein S is a reductase in vitro. Biochemist 52, 3051–3061. doi: 10.1021/bi4001358, PMID: 23566202 PMC3675161

[ref53] Lubin J. Lewis W. Gilbert N. Weimer C. Almagro-Moreno S. Boyd E.. (2015). Host-like carbohydrates promote bloodstream survival of *Vibrio vulnificus* in vivo. Infect. Immun. 83, 3126–3136. doi: 10.1128/IAI.00345-15, PMID: 26015477 PMC4496609

[ref54] Lv M. Chen Y. Liao L. Liang Z. Shi Z. Tang Y.. (2018). Fis is a global regulator critical for modulation of virulence factor production and pathogenicity of *Dickeya zeae*. Sci. Rep. 8:341. doi: 10.1038/s41598-017-18578-2, PMID: 29321600 PMC5762655

[ref55] Makino K. Oshima K. Kurokawa K. Yokoyama K. Uda T. Tagomori K.. (2003). Genome sequence of *Vibrio parahaemolyticus*: a pathogenic mechanism distinct from that of *V. cholerae*. Lancet 361, 743–749. doi: 10.1016/S0140-6736(03)12659-1, PMID: 12620739

[ref56] Mallik P. Pratt T. S. Beach M. B. Bradley M. D. Undamatla J. Osuna R. (2004). Growth phase-dependent regulation and stringent control of fis are conserved processes in enteric bacteria and involve a single promoter (fis P) in *Escherichia coli*. J. Bacteriol. 186, 122–135. doi: 10.1128/JB.186.1.122-135.2004, PMID: 14679232 PMC303451

[ref57] McCarter L. L. (1998). OpaR, a homolog of *Vibrio harveyi* LuxR, controls opacity of *Vibrio parahaemolyticus*. J. Bacteriol. 180, 3166–3173. doi: 10.1128/JB.180.12.3166-3173.1998, PMID: 9620967 PMC107818

[ref58] McCarter L. (1999). The multiple identities of *Vibrio parahaemolyticus*. J. Mol. Microbiol. Biotechnol. 1, 51–57.PMID: 10941784

[ref59] McCarter L. Hilmen M. Silverman M. (1988). Flagellar dynamometer controls swarmer cell differentiation of *V. parahaemolyticus*. Cell 54, 345–351. doi: 10.1016/0092-8674(88)90197-3, PMID: 3396074

[ref60] McCarter L. L. Wright M. E. (1993). Identification of genes encoding components of the swarmer cell flagellar motor and propeller and a sigma factor controlling differentiation of *Vibrio parahaemolyticus*. J. Bacteriol. 175, 3361–3371. doi: 10.1128/jb.175.11.3361-3371.1993, PMID: 8501040 PMC204733

[ref61] McDonald N. DeMeester K. Lewis A. Grimes C. Boyd E. (2018). Structural and functional characterization of a modified legionaminic acid involved in glycosylation of a bacterial lipopolysaccharide. J. Biol. Chem. 293, 19113–19126. doi: 10.1074/jbc.RA118.004966, PMID: 30315110 PMC6295735

[ref62] McLeod S. M. Aiyar S. E. Gourse R. L. Johnson R. C. (2002). The C-terminal domains of the RNA polymerase alpha subunits: contact site with Fis and localization during co-activation with CRP at the *Escherichia coli* proP P2 promoter. J. Mol. Biol. 316, 517–529. doi: 10.1006/jmbi.2001.5391, PMID: 11866515

[ref63] Miller M. B. Bassler B. L. (2001). Quorum sensing in bacteria. Annu. Rev. Microbiol. 55, 165–199. doi: 10.1146/annurev.micro.55.1.165, PMID: 11544353

[ref64] Miller M. B. Skorupski K. Lenz D. H. Taylor R. K. Bassler B. L. (2002). Parallel quorum sensing systems converge to regulate virulence in *Vibrio cholerae*. Cell 110, 303–314. doi: 10.1016/S0092-8674(02)00829-2, PMID: 12176318

[ref65] Miller K. A. Tomberlin K. F. Dziejman M. (2019). Vibrio variations on a type three theme. Curr. Opin. Microbiol. 47, 66–73. doi: 10.1016/j.mib.2018.12.001, PMID: 30711745 PMC6541499

[ref66] Monteiro L. M. O. Sanches-Medeiros A. Westmann C. A. Silva-Rocha R. (2020). Unraveling the complex interplay of Fis and IHF through synthetic promoter engineering. Front. Bioeng. Biotechnol. 8:510. doi: 10.3389/fbioe.2020.00510, PMID: 32626694 PMC7314903

[ref67] Münch R. Hiller K. Barg H. Heldt D. Linz S. Wingender E.. (2003). PRODORIC: prokaryotic database of gene regulation. Nucleic Acids Res. 31, 266–269. doi: 10.1093/nar/gkg037, PMID: 12519998 PMC165484

[ref68] Nair G. B. Ramamurthy T. Bhattacharya S. K. Dutta B. Takeda Y. Sack D. A. (2007). Global dissemination of *Vibrio parahaemolyticus* serotype O3:K6 and its serovariants. Clin. Microbiol. Rev. 20, 39–48. doi: 10.1128/CMR.00025-06, PMID: 17223622 PMC1797631

[ref69] Nealson K. H. Platt T. Hastings J. W. (1970). Cellular control of the synthesis and activity of the bacterial luminescent system. J. Bacteriol. 104, 313–322. doi: 10.1128/jb.104.1.313-322.1970, PMID: 5473898 PMC248216

[ref70] O’Boyle N. Boyd A. (2014). Manipulation of intestinal epithelial cell function by the cell contact-dependent type III secretion systems of *Vibrio parahaemolyticus*. Front. Cell. Infect. Microbiol. 3:114. doi: 10.3389/fcimb.2013.00114, PMID: 24455490 PMC3887276

[ref71] Osuna R. Lienau D. Hughes K. T. Johnson R. C. (1995). Sequence, regulation, and functions of fis in *Salmonella typhimurium*. J. Bacteriol. 177, 2021–2032. doi: 10.1128/jb.177.8.2021-2032.1995, PMID: 7536730 PMC176845

[ref72] Ouafa Z. A. Reverchon S. Lautier T. Muskhelishvili G. Nasser W. (2012). The nucleoid-associated proteins H-NS and FIS modulate the DNA supercoiling response of the pel genes, the major virulence factors in the plant pathogen bacterium *Dickeya dadantii*. Nucleic Acids Res. 40, 4306–4319. doi: 10.1093/nar/gks014, PMID: 22275524 PMC3378864

[ref73] Philippe N. Alcaraz J. P. Coursange E. Geiselmann J. Schneider D. (2004). Improvement of pCVD442, a suicide plasmid for gene allele exchange in bacteria. Plasmid 51, 246–255. doi: 10.1016/j.plasmid.2004.02.003, PMID: 15109831

[ref74] Prigent-Combaret C. Zghidi-Abouzid O. Effantin G. Lejeune P. Reverchon S. Nasser W. (2012). The nucleoid-associated protein Fis directly modulates the synthesis of cellulose, an essential component of pellicle-biofilms in the phytopathogenic bacterium *Dickeya dadantii*. Mol. Microbiol. 86, 172–186. doi: 10.1111/j.1365-2958.2012.08182.x, PMID: 22925161

[ref75] Prouty M. G. Correa N. E. Klose K. E. (2001). The novel sigma54- and sigma28-dependent flagellar gene transcription hierarchy of *Vibrio cholerae*. Mol. Microbiol. 39, 1595–1609. doi: 10.1046/j.1365-2958.2001.02348.x, PMID: 11260476

[ref76] Rutherford S. T. van Kessel J. C. Shao Y. Bassler B. L. (2011). AphA and LuxR/HapR reciprocally control quorum sensing in vibrios. Genes Dev. 25, 397–408. doi: 10.1101/gad.2015011, PMID: 21325136 PMC3042162

[ref77] Schneider D. A. Ross W. Gourse R. L. (2003). Control of rRNA expression in *Escherichia coli*. Curr. Opin. Microbiol. 6, 151–156. doi: 10.1016/S1369-5274(03)00038-9, PMID: 12732305

[ref78] Schneider R. Travers A. Kutateladze T. Muskhelishvili G. (1999). A DNA architectural protein couples cellular physiology and DNA topology in *Escherichia coli*. Mol. Microbiol. 34, 953–964. doi: 10.1046/j.1365-2958.1999.01656.x, PMID: 10594821

[ref79] Shao Y. Feldman-Cohen L. Osuna R. (2008). Functional characterization of the *Escherichia coli* Fis-DNA binding sequence. J. Mol. Biol. 376, 771–785. doi: 10.1016/j.jmb.2007.11.101, PMID: 18178221 PMC2292415

[ref80] Simpson C. Petersen B. Haas N. Geyman L. Lee A. Podicheti R.. (2021). The quorum-sensing systems of *Vibrio campbellii* DS40M4 and BB120 are genetically and functionally distinct. Environ. Microbiol. 23, 5412–5432. doi: 10.1111/1462-2920.15602, PMID: 33998118 PMC8458232

[ref81] Skoko D. Yoo D. Bai H. Schnurr B. Yan J. McLeod S.. (2006). Mechanism of chromosome compaction and looping by the *Escherichia coli* nucleoid protein Fis. J. Mol. Biol. 364, 777–798. doi: 10.1016/j.jmb.2006.09.043, PMID: 17045294 PMC1988847

[ref82] Steen J. A. Harrison P. Seemann T. Wilkie I. Harper M. Adler B.. (2010). Fis is essential for capsule production in *Pasteurella multocida* and regulates expression of other important virulence factors. PLoS Pathog. 6:e1000750. doi: 10.1371/journal.ppat.1000750, PMID: 20140235 PMC2816674

[ref83] Stewart B. J. McCarter L. L. (2003). Lateral flagellar gene system of *Vibrio parahaemolyticus*. J. Bacteriol. 185, 4508–4518. doi: 10.1128/JB.185.15.4508-4518.2003, PMID: 12867460 PMC165745

[ref84] Su Y. C. Liu C. (2007). *Vibrio parahaemolyticus*: a concern of seafood safety. Food Microbiol. 24, 549–558. doi: 10.1016/j.fm.2007.01.005, PMID: 17418305

[ref85] Swift S. Downie J. Whitehead N. Barnard A. Salmond G. Williams P. (2001). Quorum sensing as a population-density-dependent determinant of bacterial physiology. Adv. Microb. Physiol. 45, 199–270. doi: 10.1016/s0065-2911(01)45005-3, PMID: 11450110

[ref86] Trimble M. J. McCarter L. L. (2011). Bis-(3'-5')-cyclic dimeric GMP-linked quorum sensing controls swarming in *Vibrio parahaemolyticus*. Proc. Natl. Acad. Sci. U. S. A. 108, 18079–18084. doi: 10.1073/pnas.1113790108, PMID: 22006340 PMC3207653

[ref87] Wang H. Liu B. Wang Q. Wang L. (2013). Genome-wide analysis of the salmonella Fis regulon and its regulatory mechanism on pathogenicity islands. PLoS One 8:e64688. doi: 10.1371/journal.pone.0064688, PMID: 23717649 PMC3662779

[ref88] Weinstein-Fischer D. Altuvia S. (2007). Differential regulation of *Escherichia coli* topoisomerase I by Fis. Mol. Microbiol. 63, 1131–1144. doi: 10.1111/j.1365-2958.2006.05569.x, PMID: 17233826

[ref89] Whitaker W. B. Parent M. A. Boyd A. Richards G. P. Boyd E. F. (2012). The *Vibrio parahaemolyticus* ToxRS regulator is required for stress tolerance and colonization in a novel orogastric streptomycin-induced adult murine model. Infect. Immun. 80, 1834–1845. doi: 10.1128/IAI.06284-11, PMID: 22392925 PMC3347455

[ref90] Whitaker W. B. Richards G. P. Boyd E. F. (2014). Loss of sigma factor RpoN increases intestinal colonization of *Vibrio parahaemolyticus* in an adult mouse model. Infect. Immun. 82, 544–556. doi: 10.1128/IAI.01210-13, PMID: 24478070 PMC3911383

[ref91] Yang H. de Souza Santos M. Lee J. Law H. T. Chimalapati S. Verdu E. F.. (2019). A novel mouse model of enteric *Vibrio parahaemolyticus* infection reveals that the type III secretion system 2 effector VopC plays a key role in tissue invasion and gastroenteritis. mBio 10, 2608–2619. doi: 10.1128/mBio.02608-19, PMID: 31848276 PMC6918077

[ref92] Zhang Y. Qiu Y. Tan Y. Guo Z. Yang R. Zhou D. (2012). Transcriptional regulation of opaR, qrr2-4 and aphA by the master quorum-sensing regulator OpaR in *Vibrio parahaemolyticus*. PLoS One 7:e34622. doi: 10.1371/journal.pone.0034622, PMID: 22506036 PMC3323551

