# Supplementary material for: Fis Connects Two Sensory Pathways, Quorum Sensing and Surface Sensing, to Control Motility in Vibrio parahaemolyticus
Source: Front Microbiol. 2021 Oct 25;12:669447. doi: 10.3389/fmicb.2021.669447 (PMC8630636; doi:10.3389/fmicb.2021.669447)
Supplement: Supplementary file 1 [file Data_Sheet_1.PDF]

Supplementary Data

Fis connects two sensory pathways, quorum sensing and surface sensing, to control motility in *Vibrio parahaemolyticus*

Tague, J.G., A. Regmi, G.J. Gregory, and E.F. Boyd

Table 1. Strains and plasmids used in this study

Strain	Genotype or description	Reference or sources
**V. parahaemolyticus**		
RIMD2210633	O3:K6 clinical isolate, Str^r	(Makino, Oshima et al. 2003)
AR2885 (△fis)	RIMD2210633, Str, △fis	This study
WBWlacZ	RIMD2210633, Str, lacZ	(Whitaker, Parent et al. 2012)
ΔfispBBRfis	Δfis harboring pBBRfis, Cm^r	This study
BHM2578 (△rpoE)	RIMD2210633, Str, △rpoE	(Haines-Menges et al., 2014)
SSK2670 (△rpoN)	RIMD2210633, Str, △rpoN	(Kalburge et al., 2017)
**Escherichia coli**		
DH5αλpir	Δlac pir	Thermo Fisher Scientific
B2155λpir	ΔdapA::emr pir, for bacterial conjugation	(Dehio and Meyer, 1997)
BL21(DE3)	Expression strain	Thermo Fisher Scientific
**Plasmids**		
pDS132	Suicide plasmid, Cm^r, sacB (sucrose intolerance); R6Kg origin	(Philippe et al., 2004)
pDSΔfis	pDS132 harboring truncated fis, Cm^r	This study
pBBR1MCS	Expression vector, lacZ promoter, Cm^r	(Kovach et al., 1994)
pBBRfis	pBBR1MCS harboring full-length fis, Cm^r	This study
pRU1064	Promoterless-gfpUV; Tet^R, Amp^R; Inc origin	(Karunakaran et al., 2005)
pMAL-c5x	Empty expression vector, TEV site; Amp^R	(Liu et al., 2013)
pMALfis	pMAL harboring fis; Amp^R	This study

Table S2: Primers used in this study

Primer name	Sequences (5'-3')	Product size (bp)
**SOE PCR**		
VP2885 SOEA	TCTAGAGCATGGCAGAACAAAAGCGT	459
VP2885 SOEB	GAACATATTCGGTCTAGCTCT	
VP2885 SOEC	AGAGCTAGACCGAATATGTTCGGCATGAACTAAGACCGGA	634
VP2885 SOED	GAGCTCCTCGAGCTTCTCATCGCCTT	
VP2885 SOEFF	GATGTCCGCCAACCCAAAAC	2,982
VP2885 SOEFR	GCTGGTGATGCAAAAGGTGG	
VPA1513mutSOEA	ACCAATAAACAACTGAATACATATTAAAATATTCATTAAATTGACT	272
VPA1513mutSOEB	agatctcgctatatatAgtctcGCGattaaAtgttcatttaaacatc	
VPA1513mutSOEC	gatgtttaaatgaacaTttaatCGCGagacTatatatagcgagatct	275
VPA1513mutSOED	CTCTCTGATAAGTCCACCGTACCGACG	
**Complementation**		
FisComp F	CGAGGATCCAGAGAAGAGCTAGACCGAAT	305
FisComp R	CACTCTAGATCCGGTCTTAGTTCATGCCGTA	
**EMSA**		
VPA1424F	ACACCGGACTGGACTTTTGT	130
VPA1424R	CCATTTGAAAAGGGATTGAGTG	
VPA1674_1F	CCATCCACTGGTAGAGGTGAG	138
VPA1674_1R	TTGAGAGCTGCTTCATAGCC	
VPA1674_2F	CGTTAATCATGATCGAAAAC	152
VPA1674_2R	AATCTACTTGTTGTTATAGCCA	
VP0063F	CATAGGTTCACCTTTAAGAA	138
VP0063R	AATTTGCGCGTGATCTCAAT	
VPA0038F	GCGTAAAGCAAACGTAAAACTG	154
VPA0038R	TTTGTGCCCCTAGTTATTGGA	
VP2235F	CAATGGATTGTGCTGTTATTTAGC	161
VP2235R	TCACGCGAATACGCACTTTA	
VPA1550F	AGCCTGACGTTGGTAAGGTG	244
VPA1550R	TATGTGTTCCGCCTTCCTCT	
VP1932F	CTTTTCTCACAATCGTTACAGGA	229
VP1932R	GCCATTATCCCTCTATTTTCTGA	
VPA1513_1F	CTTTAATTTTTTACTCTGCCATT	147
VPA1513_1R	GCGTTTATCATAAAAATAGTC	
VPA1513_2F	CTTAATAATAGTTTTATCTATAAACAAAC	136
VPA1513_2R	GAGATTACTTGGTTATAAAAAG	
VPA1513_3F	TATGTTAAGTTTCATAAAATGG	139
VPA1513_3R	GAGCGTATATCCAAGTGG	

Table S2 continued

Qrr1F	CAG AAG GTA ACG AGA TTT TTG G	193
Qrr1R	GTT GGC TAG GTG ACC CGA	
Qrr2F	GAA GGG TCG AGA AGT ATT ATG C	337
Qrr2R	AAG TAT GAA ATA GTG TCG TAG TTA ATA TT	
Qrr3F	GGG AAC GGC GAG TTT AGT CAT	158
Qrr3R	GGT CAA TTT ATA TAA TGC AGT TAC TG	
Qrr4F	GAG CTC TCC TAG GTA CCA ACC	285
Qrr4R	CTC TAG ATA AAG CAC GAT GCG	
Qrr5F	GAA TAT GTT TTA CTT CCC TTA CGG	235
Qrr5R	TGA CCC TCG GCT TAG AAA GG	
**Fis purification**		
FisFWDpMAL	TGCCCATGGGAATGTTCGAACAAAATCTGACTTC	308
FisREVpMAL	CTCGGATCCGAAATCCGGTCTTAGTTCATGCCGTA	
**Reporter primers**		
PflhAF	tagatagagagagagagagagaTCGTACAAAAAGCAAAATTAAGAAAAATATAAAAC	269
PflhAR	actcattttttcttcctccaACTGCGAGGTATCTCAATC	
PlafBF	tagatagagagagagagagagaTTCTGTCGTTCTTTTCTAAGTG	456
PlafBR	actcattttttcttcctccaGAGCTTACTCCCTCTCTCATTG	
ParaBF	tagatagagagagagagagagaTGTTATCCATCCACTGGTAG	411
ParaBR	actcattttttcttcctccaGACAACTCCAACTTTTGGTC	
PnagBF	tagatagagagagagagagagaCATAGTGATATCCCTACCTGTTC	434
PnagBR	actcattttttcttcctccaTCTCATTTTGTGCCCCTAG	
PgntKF	tagatagagagagagagagagaAACTTATCCTCATAACCAAATTTG	193
PgntKR	actcattttttcttcctccaAGGTTCACCTTTAAGAATTATTGTTTG	
PscrABCF	tagatagagagagagagagagaGCGATTTCCTTCTTTATAAAG	585
PscrABCR	actcattttttcttcctccaTTTTTTCGATCCTTGTCG	
PopaRF	tagatagagagagagagagagaACTGTGCTCAATTTAGTTTG	358
PopaRR	actcattttttcttcctccaATCCATTTTCCTTGCCATTTG	
Pqrr1F	tagatagagagagagagagagaCAGAAGGTAACGAGATTTTTG	233
Pqrr1R	actcattttttcttcctccaCTAATATATCAGCATGCTTTATG	
Pqrr2F	actcattttttcttcctccaAGAAGTATTATGCATTAATCATGC	378
Pqrr2R	tagatagagagagagagagagaTTCTTTAGTGCTAAGTCATG	
Pqrr3F	tagatagagagagagagagagaAAAGTCCACTGTTTATTAATCAATC	197
Pqrr3R	actcattttttcttcctccaATTTATATAATGCAGTTACTGTGC	
Pqrr4F	tagatagagagagagagagagaAGAGCTCTCCTAGGTACC	326
Pqrr4R	actcattttttcttcctccaCTCTAGATAAAGCACGATGC	
Pqrr5F	tagatagagagagagagagagaGTCTAATAAGTTCCTACAGC	214
Pqrr5R	actcattttttcttcctccaATAGTACTAAAGCATGAGGC	

Table S3: EMSA oligonucleotides used in this study

Oligo name	Sequence	Length (bp)
Qrr3coding_WT	ccttcttaagccgag**GGTCACCTAGCCAAC**tgacgttgttagtgaa	46
Qrr3coding_mut	ccttcttaagccgag**AGTATCTTAGATAAA**tgacgttgttagtgaa	
PflhA_WT	aaaacaatcaatggatt**GTGCTGTTATTTAGC**tttgagtcattattttgg	50
PflhA_mut	aaaacaatcaatggatt**ATCATGTTATATAGA**tttgagtcattattttgg	
PlafB_WT	taaaacaccaaaatatgcc**GAGTTAAAAGATACC**aaaaatcgatgtactt	50
PlafB_mut	taaaacaccaaaatattcc**TACTTGCCATGTACA**aaaaatcgatgtactt	
PscrA_WT	ctactttagatctcgctatatat**GGTCTAAAAATTAAC**tgttcatttaaa	50
PscrA_mut	ctactttagatctcgctatatat**AGTATAGGGAGTAAA**tgttcatttaaa	
PgntK_WT	gataacat**GAATAAGATAAACCC**atattttaaagtgaattcaaaaattga	50
PgntK_mut	gataacat**AAAATAGCCAATCCA**atattttaaagtgaattcaaaaattga	
ParaB_WT	ttttt**GTCCATAAACTTAGC**aaccctatttatgggataacccac	44
ParaB_mut	ttttt**ATCACTCCTCGTAGA**aaccctatttatgggataacccac	

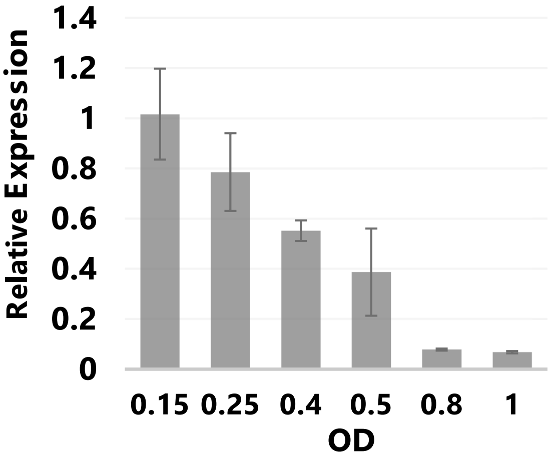

Figure S1. Expression analysis of *fis*. Examination of *V. parahaemolyticus* RIMD2210633 fis expression across the growth curve in LBS at 37˚C aerobically

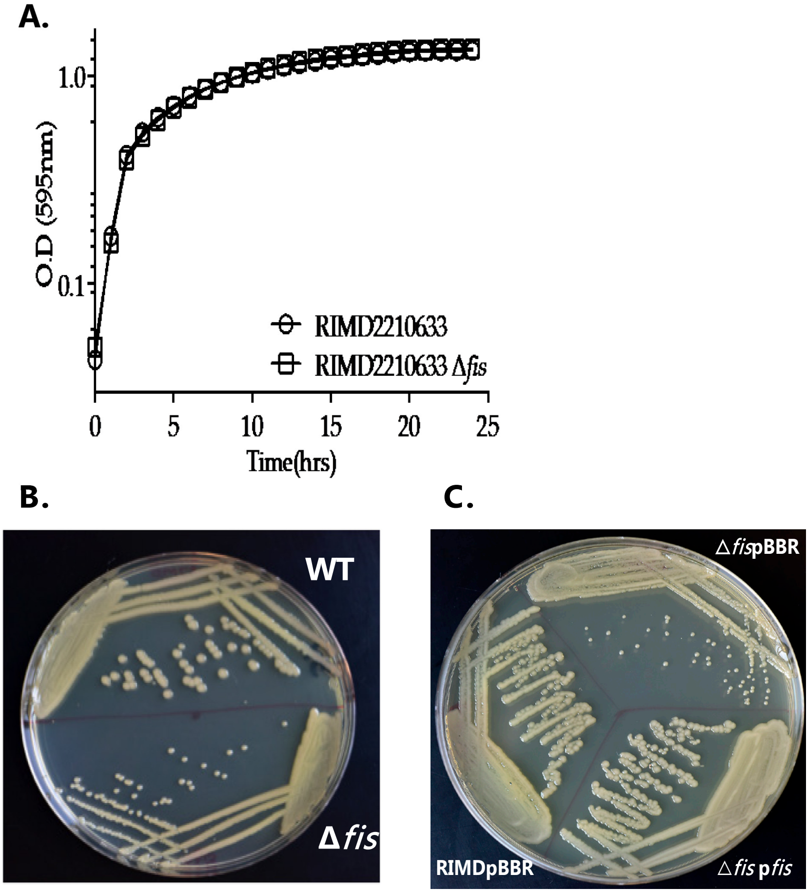

Figure S2. Growth of Δ*fis* in liquid and on solid media. A. Growth of RIMD2210633 and Δ*fis* in LBS for 24 hrs across the growth curve. No polar growth defect is observed. **B.** RIMD2210633 and Δ*fis* streaked for isolation on LBS plate. Growth defect observed for Δ*fis* mutant after incubation for 16 hours at 37˚C. **C.** Complementation of Δ*fis* with a functional copy of *fis*.

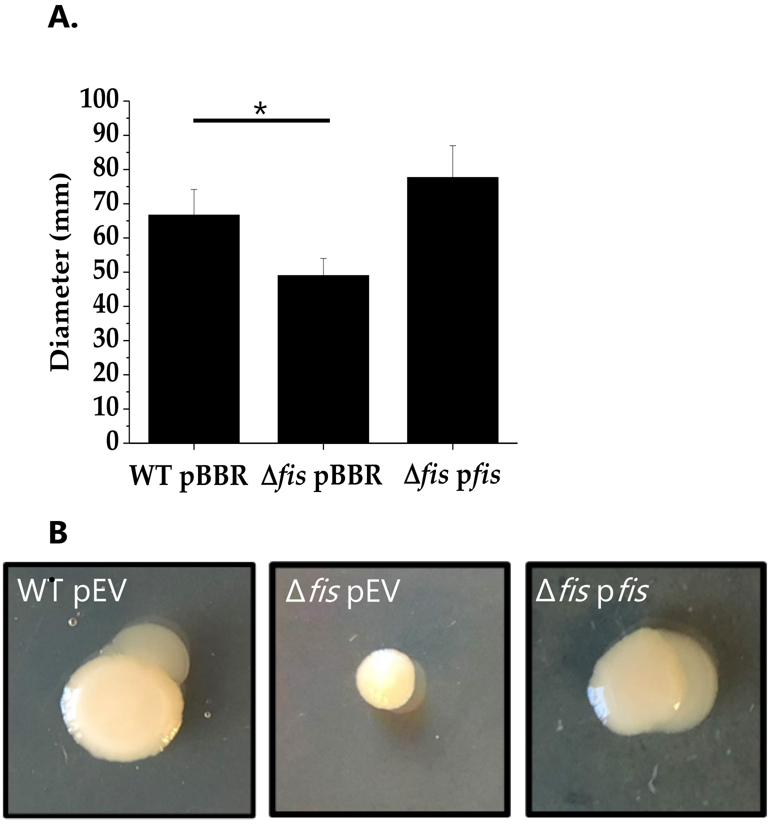

Figure S3. Complementation with pBBR*fis* rescues motility defect. A. Complementation swimming assay using pBBR1MCS empty vector (pBBR) and containing a functional copy of *fis* upstream of an IPTG inducible promoter (p*fis)* conjugated into wild type and Δ*fis* mutant. Means and standard deviation of three biological replicates plotted. Student's t-test was conducted to obtain the *P* values (*, P < 0.05). **B.** Swarming complementation assay performed using same strains as mentioned above. Pictures represent a sample of three biological replicates performed in triplicate.

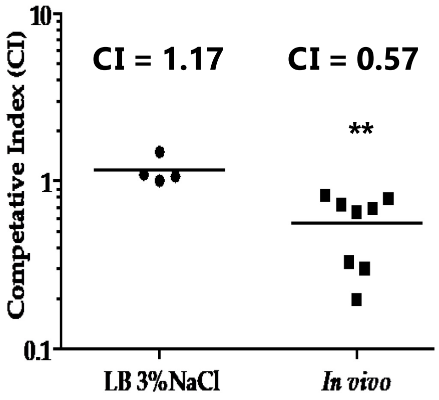

FIG S4. ***In vivo* colonization competition** A 1:1 mixed culture of WBWlacZ strain and Δ*fis* was used as the inoculum. CFU count was determined using a blue/white colony screen. Using same bacterial mixed culture, *in vitro* competition assays between WBWlacZ and Δ*fis* strain were conducted in LBS. Competitive index (CI) was calculated as CI = ratio out $_{(\Delta fis/\mathrm{WBWlacZ})}$/ratio in $_{(\Delta fis/\mathrm{WBWlacZ})}$. WBWlacZ outcompetes Δ*fis*, <1.00, and Δ*fis* outcompetes WBWlacZ when >1.00 One sample t-test was conducted to determine the P-value (*, $P < 0.05$; **, $P < 0.01$).
